# Genomic insights into the pathogenicity and environmental adaptability of *Enterococcus hirae* R17 isolated from pork offered for retail sale

**DOI:** 10.1002/mbo3.514

**Published:** 2017-08-10

**Authors:** Zixin Peng, Menghan Li, Wei Wang, Hongtao Liu, Séamus Fanning, Yujie Hu, Jianzhong Zhang, Fengqin Li

**Affiliations:** ^1^ Key Laboratory of Food Safety Risk Assessment Ministry of Health China National Center for Food Safety Risk Assessment Beijing China; ^2^ State Key Laboratory of Infectious Disease Prevention and Control Collaborative Innovation Center for Diagnosis and Treatment of Infectious Diseases National Institute for Communicable Disease Control and Prevention Chinese Center for Disease and Prevention Beijing China; ^3^ Institute of Geographic Sciences and Natural Resources Research Chinese Academy of Sciences Beijing China; ^4^ UCD‐Centre for Food Safety School of Public Health Physiotherapy and Sports Science University College Dublin Belfield Dublin Ireland

**Keywords:** drug‐resistance gene, *Enterococcus hirae*, genomic island, genomic plasticity, mobile genetic element, virulence gene

## Abstract

Genetic information about *Enterococcus hirae* is limited, a feature that has compromised our understanding of these clinically challenging bacteria. In this study, comparative analysis was performed of *E. hirae* R17, a daptomycin‐resistant strain isolated from pork purchased from a retail market in Beijing, China, and three other enterococcal genomes (*Enterococcus faecium* DO,* Enterococcus faecalis* V583, and *E. hirae* ATCC ^™^9790). Some 1,412 genes were identified that represented the core genome together with an additional 139 genes that were specific to *E. hirae* R17. The functions of these R17 strain‐specific coding sequences relate to the COGs categories of *carbohydrate transport and metabolism* and *transcription*, a finding that suggests the carbohydrate utilization capacity of *E. hirae* R17 may be more extensive when compared with the other three bacterial species (spp.). Analysis of genomic islands and virulence genes highlighted the potential that horizontal gene transfer played as a contributor of variations in pathogenicity in this isolate. Drug‐resistance gene prediction and antibiotic susceptibility testing indicated *E. hirae* R17 was resistant to several antimicrobial compounds, including bacitracin, ciprofloxacin, daptomycin, erythromycin, and tetracycline, thereby limiting chemotherapeutic treatment options. Further, tolerance to biocides and metals may confer a phenotype that facilitates the survival and adaptation of this isolate against food preservatives, disinfectants, and antibacterial coatings. The genomic plasticity, mediated by IS elements, transposases, and tandem repeats, identified in the *E. hirae* R17 genome may support adaptation to new environmental niches, such as those that are found in hospitalized patients. A predicted transmissible plasmid, pRZ1, was found to carry several antimicrobial determinants, along with some predicted pathogenic genes. These data supported the previously determined phenotype confirming that the foodborne *E. hirae* R17 is a multidrug‐resistant pathogenic bacterium with evident genome plasticity and environmental adaptability.

## 1 INTRODUCTION

*Enterococcus* spp. are opportunistic pathogens that are associated with nosocomial‐ and community‐invasive infections (Arias & Murray, 2012). These bacteria can acquire and disseminate genes that confer resistance to a variety of antimicrobial compounds including heavy metals as well as virulence factors, mediated by horizontal gene transfer (HGT) (Bellanger, Payot, Leblond‐Bourget, & Guedon, 2014; Hollenbeck & Rice, 2012; Palmer, Kos, & Gilmore, 2010; Pasquaroli et al., 2014). Acquired resistance genes can encode aminoglycoside‐modifying enzymes leading to high‐level gentamicin and streptomycin resistance phenotypes (Fair & Tor, 2014; Padmasini, Padmaraj, & Ramesh, 2014; Soleimani, Aganj, Ali, Shokoohizadeh, & Sakinc, 2014). A link has been reported between the extensive use of antimicrobial compounds in livestock production and an increase in the frequency of isolation of multidrug‐resistant (MDR) enterococci species from farm‐ and food‐producing animals (Cauwerts, Decostere, De Graef, Haesebrouck, & Pasmans, 2007). Currently, MDR enterococcal isolates are challenging nosocomial pathogens with limited available antimicrobial therapeutic options to treat these infections (Arias, Panesso, Singh, Rice, & Murray, 2009; Ceci et al., 2015; Humphries et al., 2012; O'Driscoll & Crank, 2015; Tang et al., 2015).

Enterococci, due to their fermentative potential, were thought to have the capacity when present in food matrices to extend shelf‐life and to contribute to the improvement of both flavor and texture (Dziewit et al., 2015). However, production of toxic compounds by some of these bacteria raises concerns about the safety of these isolates in food production (Camargo et al., 2014; Choi & Woo, 2013; Franz et al., 2001). Additionally, several studies reported that enterococci can transfer resistance to even more virulent bacteria, such as *Staphylococcus aureus* (Bellanger et al., 2014; Durand, Brueckner, Sampadian, Willett, & Belliveau, 2014). Hence, the ability of enterococci to act as reservoirs of antibiotic resistance genes within the food chain poses an important food safety risk (Delpech et al., 2012; Gousia, Economou, Bozidis, & Papadopoulou, 2015; Jahan, Krause, & Holley, 2013; Pesavento, Calonico, Ducci, Magnanini, & Lo Nostro, 2014; Stensland et al., 2015). Some *Enterococcus* spp. are now regarded as opportunistic zoonotic pathogens, arising from their environmental adaptability, contributing toward resistance to unfavorable factors, such as high temperature, high salt concentration, high acid and alkali environment, along with its occurrence and detection in food and food‐producing animals (Anderson et al., 2015; Hammerum, 2012; Kelesidis, 2015; Larsen et al., 2010; Manson, Hancock, & Gilmore, 2010).

Although *Enterococcus faecalis* and *Enterococcus faecium* cause the majority of reported nosocomial infections at a frequency of 90–95% and 5–10%, respectively (Ceci et al., 2015; Kristich, Rice, & Arias, 2014), *Enterococcus hirae* have historically been associated with pathological conditions in animals (Ghosh et al., 2013; Sim et al., 2012). However, in recent decades, many cases of human infection with *E. hirae* have been reported and this bacterium has been implicated as a source of severe and life‐threatening illness, such as septicemia, endocarditis, and urinary tract infections (Alfouzan et al., 2014; Anghinah et al., 2013; Bourafa, Loucif, Boutefnouchet, & Rolain, 2015; Dicpinigaitis, De Aguirre, & Divito, 2015; Kim et al., 2014; Paosinho et al., 2016; Savini et al., 2014). Moreover, the pathogenic potential of *E. hirae* may be underappreciated due to misidentification. Limited genetic information has been published to date describing *E. hirae*, particularly clinically relevant properties (Bonacina et al., 2016; Gaechter, Wunderlin, Schmidheini, & Solioz, 2012; Katyal, Chaban, & Hill, 2016; Porcellato, Ostlie, & Skeie, 2014).

The aim of this study was to describe the genome of a daptomycin‐resistant *E. hirae* R17 recovered from a food sample, and to investigate the genetic basis underpinning its antimicrobial resistance phenotype, virulence, and environmental adaptability.

## 2 MATERIAL AND METHODS

### 2.1 *Enterococcus* detection and identification

Pork meat, sewage samples, and surface swabs of the ground, walls, and chopping board were collected from a stallholder at a free‐trade market in Beijing in 2015 and analyzed microbiologically for the presence of *Enterococcus* spp., *Salmonella* spp., *Campylobacter* spp., and *Staphylococcus* spp. A quantity of 25 g samples of pork meat was aseptically weighed and separately placed in a stomacher bag (Filtra‐Bag, VWR, Luqiao Inc., Beijing, China). Then, 225 ml of brain heart infusion broth (BHI) broth was added and samples were homogenized by stomaching (BagMixer 400, Intersciences Inc., Markham, ON, Canada) for 1 min. The bags were statically incubated for 16 hr at 37°C (Jahan et al., 2013). Broth cultures for presumptive *Enterococcus* spp. were streaked onto selective isolation indoxyl‐β‐d‐glucoside (mEI) agar (Luqiao Inc., Beijing, China). Plates were then incubated at 42°C for 40–48 hr and then typical *Enterococcus* spp. colonies were screened and subsequently confirmed by VITEK 2 (bioMérieux, Marcy, l'Etoile, France), Bruker MALDI Biotyper (Germany), and 16S rDNA gene‐based sequencing as reported previously (Peng, Wang, Hu, & Li, 2016a; Peng, Wang, Hu, & Li, 2016b). All confirmed 86 *Enterococcus* spp. isolates were subjected to antimicrobial susceptibility testing.

### 2.2 Antimicrobial susceptibility testing

Susceptibility to a panel of antimicrobial agents was determined by broth microdilution, and interpreted according to the criteria based on the Clinical & Laboratory Standards Institute (CLSI) interpretive standards (CLSI, 2014). The minimum inhibitory concentrations (MIC) of 11 antimicrobial compounds were measured for 86 *Enterococcus* spp. isolates, and these included ampicillin, bacitracin, chloramphenicol, ciprofloxacin, daptomycin, erythromycin, high‐level streptomycin, high‐level gentamicin, penicillin, tetracycline, and vancomycin. The breakpoint for bacitracin susceptibility was defined as an MIC ≤ 32 μg/ml (Tran, Munita, & Arias, 2015). *Enterococcus faecalis* ATCC^™^29212 was used as a control microorganism for these experiments.

### 2.3 Complete genome sequencing, assembly, and annotation

The complete genome sequence was determined for *E. hirae* R17, and published previously (Peng et al., 2016a). The genome of *E. hirae* R17 was sequenced using the Pacific Biosciences RS II sequencing platform (Pacific Biosciences, Menlo Park, CA, USA). Single‐molecule real‐time (SMRT^®^) sequencing was conducted using the C4 sequencing chemistry and P6 polymerase with one SMRT^®^ cell. De novo assembly of the PacBio reads were carried out using continuous long reads (CLR) following the hierarchical genome assembly process (HGAP) workflow (PacBioDevNet; Pacific Biosciences) as available in SMRT^®^ Analysis v2.3 (Chin et al., 2013). The functions of predicted proteins were annotated based on homologs (using SwissProt, http://www.uniprot.org/uniprot/), and clusters of ortholog groups were determined (using COG, http://www.ncbi.nlm.nih.gov/COG/). The NCBI Prokaryotic Genome Annotation Pipeline (PGAP) was employed to identify coding sequences (CDS) based on the best‐placed reference protein set and GeneMarkS+. The complete genome sequence of *E. hirae* R17 was deposited in GenBank under the accession number CP015516 (chromosome) and CP015517 (plasmid pRZ1).

### 2.4 Comparative genomic analysis

Three complete and annotated genomes of *E. faecium* DO (accession number from CP003583 to CP003586), *E. faecalis* V583 (accession number from AE016830 to AE016833), and *E. hirae* ATCC^™^9790 (accession number CP003504.1 and HQ724512.1) were available from GenBank and used as reference sequences in this study. The GC content of each sequence was calculated using the GC‐Profile web server (Gao & Zhang, 2006). Orthologs between all three reference genomes and *E. hirae* R17 were identified using OrthoMCL software by reference to their protein sets (Fischer et al., 2011). Significant similarity was defined as E‐value ≤1e‐05, the length of the protein alignment ≥60%, and amino acid sequence identity ≥50%, and the best hits were selected and further analyzed.

### 2.5 *Sequence analysis of the E. hirae* R17 genome

CRISPR loci were identified in the *E. hirae* R17 genome using CRISPRfinder (http://crispr.u-psud.fr/Server/CRISPRfinder.php) (Grissa, Vergnaud, & Pourcel, 2007). The insertion sequences (IS) of this bacterium were identified initially using the ISfinder database (http://www-is.biotoul.fr/is.html), and annotated transposons were searched against the NCBI nucleotide sequence database (Siguier, Varani, Perochon, & Chandler, 2012). Repeat sequences were identified using Tandem Repeats Finder Program (http://tandem.bu.edu/trf/trf.html) (Benson, 1999). Genomic islands (GIs) were searched using IslandViewer (http://www.pathogenomics.sfu.ca/islandviewer/browse/) (Dhillon et al., 2015). The antimicrobial resistance genes were predicted by the Antibiotic Resistance Genes Database (ARDB) (http://ardb.cbcb.umd.edu/) (Liu & Pop, 2009), the Comprehensive Antibiotic Resistance Database (CARD) (https://card.mcmaster.ca/analyze) (McArthur et al., 2013), and ResFinder 2.1 (https://cge.cbs.dtu.dk/services/ResFinder/) (Zankari et al., 2012). Virulence factors were identified using the virulence factor database (VFDB) (http://www.mgc.ac.cn/VFs/main.htm) (Chen, Zheng, Liu, Yang, & Jin, 2016) and VirulenceFinder 1.5 (https://cge.cbs.dtu.dk/services/VirulenceFinder/) (Kleinheinz, Joensen, & Larsen, 2014). PathogenFinder 1.1 (https://cge.cbs.dtu.dk/services/PathogenFinder/) was used to distinguish pathogenic from nonpathogenic bacteria using the whole genome sequence data of *E. hirae* R17 (Cosentino, Voldby Larsen, Moller Aarestrup, & Lund, 2013). The BacMet database (http://bacmet.biomedicine.gu.se/) was used to predict the antibacterial biocide‐ and metal resistance encoding genes (Pal, Bengtsson‐Palme, Rensing, Kristiansson, & Larsson, 2014). For 16S rRNA phylogenetic analyses and average nucleotide identity (ANI), all 28 enterococcal 16S rRNA and 30 enterococcal whole genomic sequences were obtained from the NCBI database. Phylogenetic analysis of 28 16S rRNA sequences, belonging to 10 different spp., including *Enterococcus avium*,* Enterococcus durans*,* Enterococcus faecalis*,* Enterococcus faecium*,* Enterococcus hirae*,* Enterococcus mundtii*,* Enterococcus pseudoavium*,* Enterococcus ratti*,* Enterococcus thailandicus*, and *Enterococcus villorum*, were aligned with ClustalW using the BLOSUM matrix and further aligned visually. Maximum likelihood phylogenetic trees were created in MEGA5.2 (Tamura et al., 2011) using the Poisson Model and 1000 bootstrap iterations. ANI analysis of 30 whole genomic sequences, belonging to 13 different spp., including *E. avium*,* Enterococcus casseliflavus*,* Enterococcus cecorum*,* E. durans*,* E. faecalis*,* E. faecium*,* Enterococcus gallinarum*,* E. hirae*,* E. mundtii*, *E. pseudoavium*,* Enterococcus raffinosus*,* E. thailandicus*, and *E. villorum*, were calculated for each pair of genomes by JSpecies (Richter & Rosselló‐Móra, 2009) using ANIb (ANI based on BLAST) method with default parameters (Konstantinidis & Tiedje, 2005). A phylogenic tree was built based on the ANI matrix using BIONJ (Gascuel, 1997) method with APE (Paradis, Claude, & Strimmer, 2004) package version 4.1 in R version 3.3.2.

## 3 RESULTS

### 3.1 Features of the *E. hirae* R17 genome

**Figure 1:**
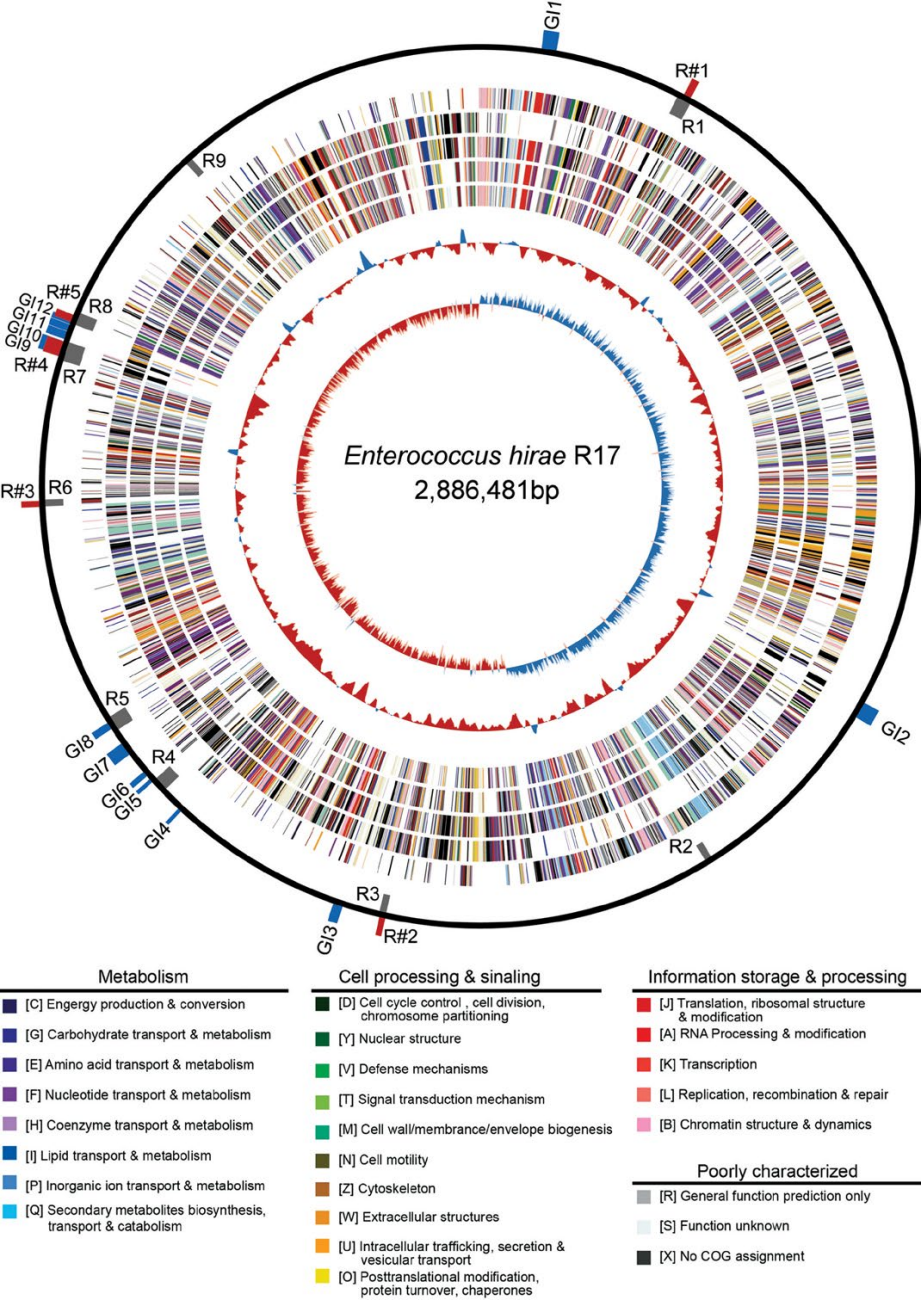
Circular map of the *Enterococcus hirae* R17 genome. Circles from outside to inside are as follows: (1) scale marks of the *E. hirae* R17 genome. The blue rectangles represent genomic islands (GI1 through GI12), gray rectangles represent genomic regions unique in *E. hirae* R17 compared to *E. hirae* ATCC ^™^9790 (R1 through R9), and red rectangles represent genomic regions unique in *E. hirae* R17 compared to *E. hirae* ATCC ^™^9790, *Enterococcus faecium* DO, and *Enterococcus faecalis* V583 (R#1 through R#5); (2) and (3) predicted proteins encoded by genes on the forward and reverse strands; (4), (5), and (6): common genes shared by *E. hirae* R17 with those of reference genomes *E. hirae* ATCC ^™^9790, *E. faecium* DO, and *E. faecalis* V583, respectively; (7) GC content percentage, above median GC content (red), less than median (blue); (8) GC skew [(G‐C)/(G+C)], values >0 (red), values <0 (blue). Gene colors indicate the COG categories to which they belong are shown below the map

An *E. hirae* strain with DAP resistance was screened, designated R17, and its complete genome sequence was determined. As shown in Table 1, the complete genome sequence of *E. hirae* R17 consisted of a single circular chromosome and one circular plasmid. The chromosome contained 2,886,481 bp with 2,431 protein‐coding open reading frames (ORFs), which represented 83.3% of the chromosome, 66 tRNAs, and 18 ribosomal rRNAs, four other noncoding RNAs, 55 pseudogenes, and one CRISPR‐*cas* sequence. The chromosome had a GC content of 36.96%, and it showed a clear GC skew at the origin of replication (Figure 1). The size of the plasmid pRZ1 was found to be 73,574 bp with a GC content of 35.57%, encoding 66 ORFs, which covered 85.9% of the plasmid, and also encoding seven pseudogenes.

**Table 1:** Comparison of *E. hirae* R17 with other *Enterococcus* spp. with complete genome sequences published

	Strain											
	*E. hirae* R17		*E. hirae* ATCC^™^9790		*E. faecium* DO				*E. faecalis* V583			
Source	Fresh pork		No source information		Endocarditis patient’ blood				Patient's blood			
GenBank	CP015516.1	CP015517.1	CP003504.1	HQ724512.1	CP003583.1	CP003584.1	CP003585.1	CP003586.1	AE016830.1	AE016831.1	AE016832.1	AE016833.1
Status	Chromosome	Plasmid	Chromosome	Plasmid	Chromosome	Plasmid	Plasmid	Plasmid	Chromosome	Plasmid	Plasmid	Plasmid
Size (bp)	2,886,481	73,574	2,827,741	28,699	2,698,137	36,262	66,247	251,926	3,218,031	57,660	17,963	66,320
GC%	37.0	35.6	36.9	33.3	38.2	36.5	34.4	36.0	37.5	33.9	33.3	34.4
Genes	2,574	73	2,669	33	2,795	44	87	283	3,257	62	19	74
Proteins	2,431	66	2,452	0	2,703	43	85	283	3,112	62	18	72
rRNA	18	0	18	0	18	0	0	0	12	0	0	0
tRNA	66	0	71	0	62	0	2	0	68	0	0	0
Pseudogene	55	7	128	0	–	–	–	–	1	0	0	0
CRISPR‐*cas*	+		+		none				none			
Country	China		No information		USA				USA			
Release date (Modify date)	2016‐05‐12		2012‐06‐20 (2015‐08‐18)		2012‐05‐25 (2016‐04‐22)				2003‐03‐28 (2016‐04‐19)			
Reference	Peng et al. (2016a); Peng et al., (2016b)		Gaechter et al. (2012)		Qin et al. (2012)				Paulsen et al. (2003)			

### 3.2 Comparative genome analysis of *E. hirae* R17 with three other *Enterococcus* genomes

**Figure 2:**
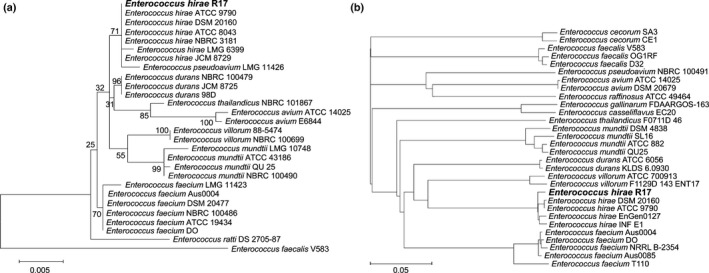
Maximum likelihood phylogenetic tree of the 16S rRNA gene sequences (a) and the whole genome phylogenetic tree constructed by average nucleotide identity (ANI) (b) of *Enterococcus hirae* R17 with other *Enterococcus* species. Bootstrap values (a) and branch lengths represent the distance (b) based on ANI and are labeled at the nodes of figures

A 16S rRNA and an ANI phylogenetic tree describing the species belonging to genus *Enterococcus* were constructed based on 16S rRNA sequences and whole genomic sequences, respectively, and in which species within the same taxonomic groups were tightly clustered (Figure 2). For the 16S rRNA phylogenetic tree, *E. hirae* R17 and reference strain *E. hirae* ATCC^™^9790 were observed to cluster together. Although *E. hirae* R17 is considered distantly related to isolates *E. faecium* DO and *E. faecalis* V583 as shown in the phylogenetic tree, its 16S rRNA sequence was 99% identical to the sequences in those two strains. The 16S rRNA phylogenetic tree also revealed that the *E. hirae* cluster was closely related to *E. durans*, but distant from *E. faecium, E. mundtii*, and *E. faecalis* (Figure 2a). The ANI tree provides an in‐depth phylogenetic relationship at the species and strain levels (Figure 2b). The *E. hirae* cluster was closely related to *E. villorum* and *E. durans*, and then *E. faecium* and *E. mundtii*, but distant from *E. pseudoavium, E. avium, E. cecorum,* and *E. faecalis*. Within the *E. hirae* cluster, *E. hirae* ATCC^™^9790 was closest to *E. hirae* DSM 20160, and *E. hirae* EnGen0127 and INF E1 were clustered together. The above four *E. hirae* strains were closely related to *E. hirae* R17.

Comparative genome analysis was performed for the genomes of *E. hirae* R17, *E. hirae* ATCC^™^9790, *E. faecium* DO, and *E. faecalis* V583 and revealed that the genome sizes varied substantially from 2.70 Mb (DO) to 3.22 Mb (V583), and the number of genes ranged from 2,574 (R17) to 3,257 (V583) (Table 1). Of the four genome sequences analyzed, *E. hirae* R17 and ATCC^™^9790 alone possessed CRISPR1‐*cas* loci. Orthologs analysis indicated that a total of 2,153 genes were shared between *E. hirae* R17 and ATCC^™^9790 (Figure 1), and some 278 genes and nine genomic regions (containing more than five consecutive strain‐specific genes) were unique to *E. hirae* R17 (Table S1 and Figure 1). In total, 1,412 genes were shared across all four genomes, and 139 genes and five regions were unique in *E. hirae* R17 (Table S2 and Figure 1). The majority of these strain‐specific genes are hypothetical proteins. The inner‐ and intraspecies genome comparison indicated that gene exchanges appeared to be a common feature among the *Enterococcus* spp. Strain‐specific proteins of known function mostly belonged to the COG categories of *carbohydrate transport and metabolism, transcription*, and *cell wall/membrane/envelope biogenesis* (Table S1 and Table S2), suggesting that carbohydrate utilization and cell wall/membrane/envelope functions of *E. hirae* R17 may be different from the other three bacteria.

Two *E. hirae* R17‐specific genomic regions (genomic region #4 [located from 2,304,674 to 2,319,265 bp] and #5 [located from 2,336,013 to 2,343,132 bp] shown in Table S2) were identified as putative GI, which may be the result of HGT because both regions contain transposases that mapped closely together. In particular, region #4 consisted of 10 hypothetical proteins, one for cell wall/membrane/envelope biogenesis, one for signal transduction mechanisms, and one for replication, recombination and repair. Region #5 contained glycosyl transfer‐related genes, and may be involved in cell wall/membrane/envelope biogenesis.

### 3.3 Genomic islands identified in *E. hirae* R17

GIs differ in GC content, codon usage, and *k*‐mer frequencies from the rest of the genome due to their origins (Dhillon et al., 2015). A total of 13 areas of the sequence including 122,195 bp were predicted as GIs in *E. hirae* R17, of which 12 GIs were loci on the chromosome and one was found on the plasmid (Table S3). Genomic islands five and six may be a single GI as they are located very close together (separated by only 4,263 bp). Similarly, GI‐9, ‐10, ‐11, and ‐12 likely form a single GI because their encoding ORFs are close together.

As shown in Table S3, many of the hypothetical proteins, pseudogenes, and transposon‐related proteins were found in the 12 predicted GIs on the chromosome. Genomic island seven contained 10 hypothetical proteins, containing four possible pseudo genes, two predicted transposases, and one putative transposon, Tn*552* DNA‐invertase bin3. In addition to seven hypothetical proteins, GI‐6 contained the HTH‐type transcriptional repressor RghR, a toxin‐encoding gene with ambiguous function, and one tyrosine recombinase XerC‐like gene. On GI‐2 and ‐4, four and six genes were identified, respectively, encoding hypothetical proteins. Seven genes including six hypothetical proteins and one uncharacterized phage‐related protein Lin1259/Lin1739 were identified on GI‐5. Genomic islands 9, 10, 11, and 12 contained 29 ORFs, including 15 that were hypothetical proteins, one transposase, and one viral‐enhancing factor. The hyaluronan synthase gene (AND73241.1), was considered to be a virulence factor in clinical isolates of *E. faecium* (Arias et al., 2009), and this gene was also located on GI‐10. The dispersion of transposases identified on these GIs may relate to their acquisition by HGT.

Genomic islands three and eight consisted of six and five known genes, respectively. Genomic island three contained three galactosidase‐related proteins, two regulatory proteins, and one histidine kinase. Three potassium‐transporting related proteins, one sensor protein, and a tagatose aldolase were located in GI‐8. There were 25 ribosomal proteins in GI‐1, a common feature among the genomic islands of foodborne enterococcal genomes previously sequenced (Bonacina et al., 2016).

The sole GI identified on plasmid pRZ1 contained erythromycin‐ and bacitracin‐resistance‐related genes, and these may function to promote *E. hirae* R17 colonization and infection under selection. The two transposases located on this island suggested that the antibiotic resistance genes may have been acquired by HGT with the potential to be mobilized *en bloc*.

### 3.4 Virulence gene and pathogenicity prediction

To identify possible virulence genes in the *E. hirae* R17 genome, the virulence factors listed in the VFDB were aligned to ORF protein sequences using BLASTP and filtered with 70% identity and 70% match length. A total of 13 virulence factors were identified (Table S4) as follows: LacI family sugar‐binding transcriptional regulator BopD (protein_id:AND71552.1), undecaprenyl diphosphate synthase UppS (AND72745.1), phosphopyruvate hydratase Eno (AND73037.1), ATP‐dependent Clp protease proteolytic subunit (AND73144.1), phosphatidate cytidylyltransferase EFAU085_01747 (AND72744.1), putative dTDP‐glucose‐4, 6‐dehydratase RmlB (AND71992.1), glucose‐1‐phosphate thymidylytransferase RmlA (AND71990.1), glyceraldehyde‐3‐phosphate dehydrogenase Plr/GapA (AND73040.1), glucose‐1‐phosphate uridylyltransferase SMU.322c (AND72989.1), two‐component response regulator LisR/LisK (AND72094.1), glucose‐1‐phosphate uridylyltransferase SMU.322c (AND73197.1), periplasmic solute‐binding protein EfaA (AND73314.1), and translation elongation factor Tu EF‐Tu (AND72142.1). These factors are all well‐characterized virulence determinants in *E. faecium* and other pathogens such as *Listeria monocytogenes*,* Streptococcus pneumonia*,* Streptococcus mutans*,* Streptococcus sanguinis*, and *Mycoplasma mycoides*.

In addition to the high identity virulence factors mentioned above, the virulence factors filtered with 50% identity and 50% match length are listed in Table S4. A total of four well‐studied virulence factors were identified at this cut‐off: a capsular polysaccharide (AND72952.1) with 67.49% identity to that of *Bacillus cereus* ATCC^™^10987, two biofilm‐associated Ebp pili proteins (AND71603.1 and AND71600.1) with 58.87 and 57.5% identity, respectively, to proteins of *E. faecalis* OG1RF and *E. faecalis* D32, and a hyaluronidase (AND72041.1) with 56.15% identity to that of *E. faecalis* V583.

The analysis of the plasmid pRZ1 revealed a 0.937 probability that this may be considered a human pathogenic plasmid, and the matched protein (BAF76914) was from the human pathogen *Staphylococcus aureus* subsp. *aureus* Mu3, which functions in plasmid recombination. The probability of the chromosome of *E. hirae* R17 being a human pathogen was 0.696, and three pathogenicity hits matched the SSU ribosomal protein S19P (ABP89047) of *Streptococcus suis* 05ZYH33, a conserved protein (CAR86734) of *Lactobacillus rhamnosus* GG, and ribosomal protein L29 (AAO080083) of *E. faecalis* V583.

### 3.5 Resistance to antimicrobial agents

Using the genome to query databases containing antibiotic resistance‐encoding genes showed that the *E. hirae* R17 sequence possessed 33 resistance‐encoding genes. Of these, 27 of these genes were located to the chromosome and six to the plasmid (Table 2). Based on these data, *E. hirae* R17 could confer resistance to several antimicrobial compounds, including beta‐lactam antibiotics, lincosamide, peptide antibiotics, pleuromutilin, polymyxin, streptogramin, tetracycline, and others. Among the 11 classes of antibiotics tested, *E. hirae* R17 expressed resistance to bacitracin (MIC>512 μg/ml), ciprofloxacin (MIC>8 μg/ml), daptomycin (DAP, MIC=8 μg/ml), erythromycin (MIC>8 μg/ml), and tetracycline (MIC>32 μg/ml).

**Table 2:** Antibiotic resistance genes in *E. hirae* R17

Encoded Protein	Location	Protein ID	Function or resistant to
IsaA	Chromosome	AND71848.1	Efflux pump conferring resistance to lincosamide, streptogramin, and pleuromutilin
ParY/GyrB	Chromosome	AND71321.1	Aminocoumarin, ciprofloxacin
MecC	Chromosome	AND72540.1	Beta‐lactam antibiotics
EF‐Tu	Chromosome	AND71360.1	Kirromycin
ImrC	Chromosome	AND73497.1	Efflux pump conferring resistance to lincosamide
AdeC	Chromosome	AND73730.1	Efflux pump conferring resistance to tetracycline
MprF	Chromosome	AND72143.1	Peptide antibiotic, daptomycin
PBP2b	Chromosome	AND72082.1	Beta‐lactam antibiotics
DfrE	Chromosome	AND72186.1	Trimethoprim
RpoB	Chromosome	AND73611.1	Rifampin
ImrD	Chromosome	AND73496.1	Lincosamide
MprF	Chromosome	AND73546.1	Peptide antibiotic, daptomycin
PBP1b	Chromosome	AND72982.1	Beta‐lactam antibiotics
PmrE	Chromosome	AND72949.1	Polymyxin
PBP1a	Chromosome	AND72396.1	Beta‐lactam antibiotics
Tet42	Chromosome	AND72539.1	Efflux pump conferring resistance to tetracycline
PBP2x	Chromosome	AND71950.1	Beta‐lactam antibiotics
lmrB	Chromosome	AND71563.1	Efflux pump conferring resistance to lincosamide
AlaS	Chromosome	AND72410.1	Aminocoumarin
Cls	Chromosome	AND73003.1	Lipopeptide antibiotic, daptomycin
MurA	Chromosome	AND73017.1	Fosfomycin
IleS	Chromosome	AND71962.1	Mupirocin
EF‐Tu	Chromosome	AND72142.1	Kirromycin, elfamycin
AAC(6′)‐Iid	Chromosome	AND73261.1	Aminoglycoside
ArlR	Chromosome	AND72094.1	Efflux pump conferring resistance to fluoroquinolone
MprF	Chromosome	AND72343.1	Peptide antibiotic, daptomycin
Mfd	Chromosome	AND73512.1	Fluoroquinolone
BcrD	Chromosome	AND72321.1	Bacitracin
BcrD	Plasmid	AND73778.1	Bacitracin
BcrB	Plasmid	AND73779.1	Bacitracin ABC transporter permease
BcrA	Plasmid	AND73780.1	Bacitracin ABC transporter ATP‐binding protein
BcrR	Plasmid	AND73781.1	Bacitracin‐related transcriptional regulator
ErmB	Plasmid	AND73775.1	Lincosamide, macrolide, streptogramin
TetK	Plasmid	AND73772.1	Tetracycline resistance MFS efflux pump
TetT	Plasmid	AND73771.1	Tetracycline resistance ribosomal protection protein

**Figure 3:**
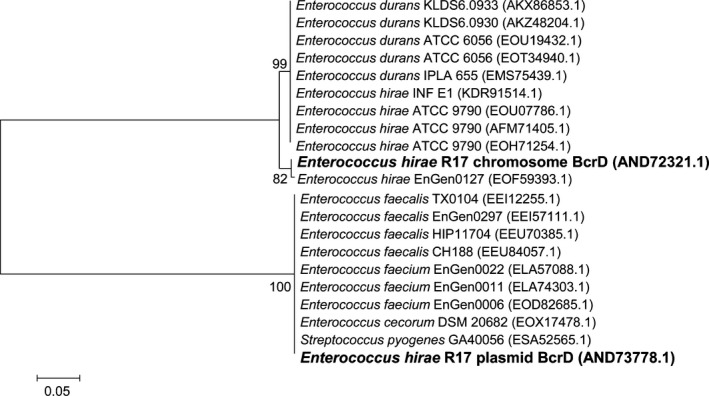
Maximum likelihood phylogenetic comparison of the BcrD locus on the chromosome and plasmid of *Enterococcus hirae* R17 compared with most identity proteins, respectively. Bootstrap values are labeled at the nodes

Interestingly, *E. hirae* R17 has two copies of the bacitracin‐resistance related gene *bcrD*, one on the chromosome and one on the plasmid. Alignments showed that the identity of the two predicted BcrD was only 54%. The most similar sequences to the BcrD from the chromosome (AND72321.1) and from the plasmid (AND73778.1) were separately used to make a phylogenetic tree, and the result showed that the BcrD on chromosome was far in phylogenetic distance from the BcrD on the plasmid (Figure 3). The chromosomal BcrD of *E. hirae* R17 was close to that of *E. hirae* and *E. durans*. However, the *bcrD* encoded on the plasmid was more similar to the genes of *E. faecium* and *E. faecalis*, suggesting it may have evolved from intraspecies HGT.

A single‐ nucleotide polymorphism was identified in the *gyrB* (*parY*) gene at position 1,129 (giving rise to a transition mutation, A→G) resulting in the substitution of an isoleucine to a valine in GyrB, a protein that plays an important role in aminocoumarin and ciprofloxacin resistance. In addition, there were three copies of the DAP‐resistance‐related *mprF* gene and one copy of the *cls* gene on the *E. hirae* R17 chromosome, which play roles in lysylphosphatidylglycerol and cardiolipin synthesis, respectively (Diaz et al., 2014).

### 3.6 Antibacterial biocide and metal resistance genes

The predicted antibacterial biocide and metal resistance genes are listed in Table 3. Potential resistance genes related to hydrochloric acid, hydrogen peroxide, copper, silver, iron, and selenium were encoded on the chromosome, and the resistance genes related to ethidium bromide, sodium dodecyl sulfate, and tetraphenylphosphonium, as well as the cetrimide resistance‐related gene *lmrS* were found on the plasmid. Interestingly, the predicted *lmrS* gene overlapped the tetracycline resistance MFS efflux pump gene *tet*(K), suggesting similarity in the mechanism of tetracycline‐ and antibacterial biocide resistance.

**Table 3:** Biocide and metal resistance genes in *E. hirae* R17

Encoded protein	Location	Protein ID	Resistant to
GadC/XasA	Chromosome	AND71844.1	Hydrochloric acid (HCl)
SodA	Chromosome	AND72023.1	Selenium (Se), hydrogen peroxide (H_2_O_2_)
CopB	Chromosome	AND72927.1	Copper (Cu), Silver (Ag)
CopA	Chromosome	AND72928.1	Copper (Cu), Silver (Ag)
CopZ	Chromosome	AND72929.1	Copper (Cu)
CopY/TcrY	Chromosome	AND72930.1	Copper (Cu)
Dpr/Dps	Chromosome	AND73605.1	Iron (Fe), hydrogen peroxide (H_2_O_2_)
LmrS	Plasmid	AND73772.1	Tetraphenylphosphonium (TPP), Sodium dodecyl sulfate (SDS), Ethidium bromide, cetrimide (CTM)

### 3.7 Mobile genetic elements and genomic plasticity

The genome of *E. hirae* R17 contained 14 transposase genes, of which nine genes were located on the chromosome and five were located on the plasmid (Table 4). Among the 14 transposase genes, seven were predicted to be pseudogenes. Considering the sizes of the chromosome and the plasmid (chromosome, 2,886,481 bp and the plasmid pRZ1, 73,574 bp), the plasmid DNA may be more susceptible to IS element/transposase insertions. A region on the chromosome (AND73223.1–AND73237.1) that is related to GI‐9 may be the result of HGT because this sequence was found to be flanked by two IS*Efa11* transposase genes from the IS*L3* family. Interestingly, the transposase AND73254.1 on the chromosome did not match any IS sequence in the IS Finder database, and may represent a previously uncharacteristic type of IS. On the plasmid, four IS*6* family transposases were identified; two are the IS*1216E*‐type transposase and the other two are of the IS*Enfa1* type. The region flanked by the two IS*1216E*‐type transposases (AND73768.1‐AND73774.1) contained the tetracycline‐resistance gene cluster, and two IS*Enfa1*‐type transposases bordered the region (AND73777.1–AND73783.1) that contains the bacitracin‐resistance‐related genes, which indicated that the two antibiotic resistance gene clusters may have been gained by HGT from other bacteria.

**Table 4:** Mobile elements in *E. hirae* R17

Locus Tag	Protein ID	Start	End	Predicted gene/family
Chromosome				
A6P53_02125	–	472739	472994	Transposase; pseudogene
A6P53_05165	–	1155205	1156170	Transposase; pseudogene
A6P53_05625	‐	1257978	1258525	Transposase; pseudogene
A6P53_06910	–	1543140	1543598	Transposase; pseudogene
A6P53_08380	–	1866711	1867133	Transposase; pseudogene
A6P53_08435	–	1880323	1880745	Transposase; pseudogene
A6P53_10350	AND73223.1	2299459	2300754	IS*Efa11* (IS*L3* family) transposase
A6P53_10420	AND73237.1	2319680	2320975	IS*Efa11* (IS*L3* family) transposase
A6P53_10505	AND73254.1	2345697	2346704	Transposase
Plasmid				
A6P53_12990	AND73768.1	20492	21172	IS*1216E* (IS*6* family) transposase
A6P53_13025	AND73774.1	28639	29319	IS*1216E* (IS*6* family) transposase
A6P53_13040	AND73777.1	31420	32106	IS*Enfa1* (IS*6* family) transposase
A6P53_13070	AND73783.1	36139	36825	IS*Enfa1* (IS*6* family) transposase
A6P53_13110	–	46315	47581	Transposase; pseudogene

The numbers of tandem repeats (TR) identified hinted at genomic plasticity and bacterial environmental adaptation (Munita, Bayer, & Arias, 2015). Some 127 TR were identified with period sizes ranging from 6 to 441 bp on the chromosome and two tandem repeats with period sizes of 8 bp on the plasmid (Table S5). Many tandem repeats of *E. hirae* R17 were located in protein‐coding regions, and thus could be part of the mechanism for the generation of targeted gene variation.

### 3.8 Plasmid pRZ1 in *E. hirae* R17

**Figure 4:**
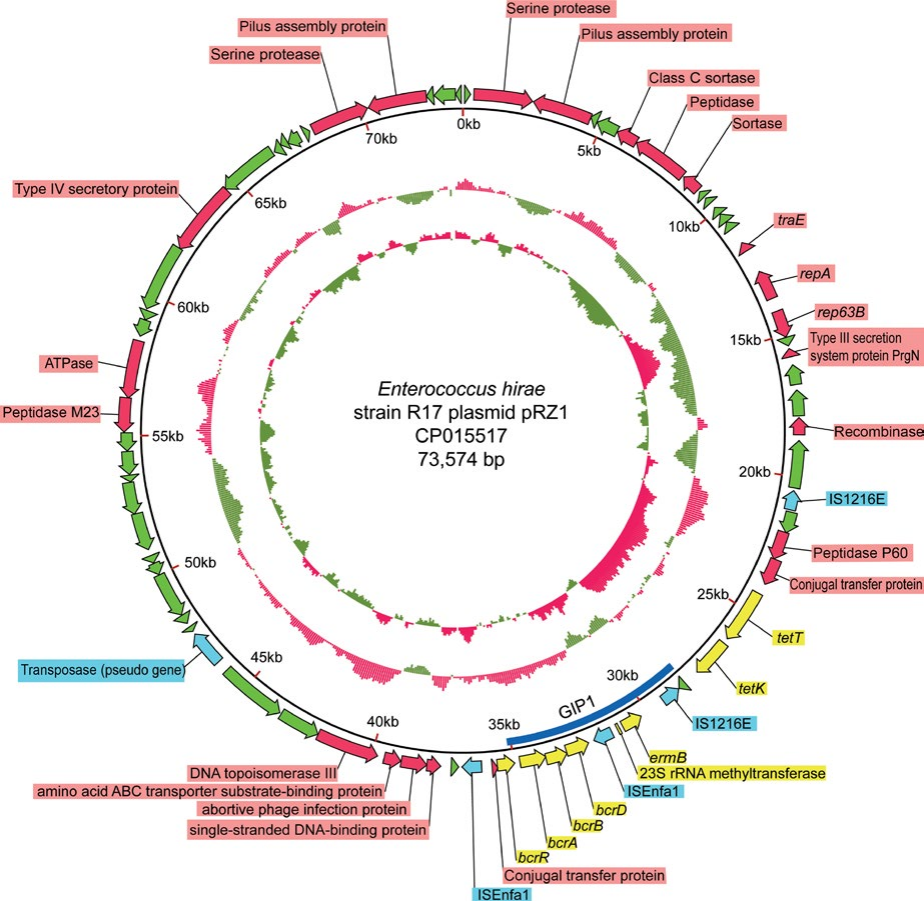
A schematic representation of the genetic map of plasmid pRZ1. Genes are denoted by arrows and are colored based on gene function: green arrows represent hypothetical proteins; blue arrows represent transposases; yellow arrows represent predicted antibiotic resistance genes; red arrows represent other encoding genes of known function; blue stripped represent genomic island (GIP1). The innermost circle presents the GC skew [(G‐C)/(G+C)], values >0 (red), values <0 (green). The outer circle represents the GC content, above median GC content (red), less than the median (green)

Alignment of the 73.6 kb length sequence of plasmid pRZ1 in NCBI using BLASTN showed the highest query cover rate was 57%, which indicated that pRZ1 was a new plasmid. The highest matched sequence found was to plasmid pDO3 from *E. faecium* DO. Of the 66 predicted ORFs in the plasmid pRZ1 sequence, most were hypothetical proteins (Figure 4). Three conjugal transfer proteins indicate that plasmid pRZ1 may be capable of conjugal transfer to a new host.

A 16.3 kb‐region (AND73768.1–AND73783.1) containing four IS*6* family elements and six genes associated with the resistance of bacitracin, macrolide antibiotics, and tetracycline (Figure 4), that showed similarity to the chromosomal sequence of *Streptococcus gallolyticus* UCN34, *S. aureus* subsp. *aureus* JS395, and *E. faecium* E506 with 44% match length, indicating that the architecture of the region was unique. Plasmid pRZ1 harbored a type III secretion system protein, PrgN (AND73763.1), and a type IV secretory protein (AND73805.1), which may contribute to the pathogenicity of the plasmid.

## 4 DISCUSSION

Enterococci are bacteria known to be involved in the dissemination of resistance to antimicrobial compounds (Munita et al., 2014). In earlier reports, *E. hirae* were found more frequently in municipal waste water, river, soil, and animals, and less frequently identified in sources such as fruits, vegetable, and food products of animal origin (Chubiz, Lee, Delaney, & Marx, 2012; Munita et al., 2014). Therefore, the isolation and identification of *E. hirae* R17 from a retail pork sample was of interest because of its MDR phenotype and potential pathogenicity. In this study, a genomic comparison between *E. hirae* R17 and other selected enterococcal reference genomes was made. GIs, virulence‐encoding genes, antimicrobial resistance genes, among others were investigated.

Genome comparison of the foodborne *E. hirae* R17 to inter‐ and intraspecies isolates allowed us to determine the phylogeny of *E. hirae* and to accurately quantify the genomic diversity between strains of the genus *Enterococcus*. The ANI phylogenetic tree provides stronger discriminatory power than the 16S rRNA tree and enables enterococcal strain differentiation. The phylogenetic relationships among *Enterococcus* strains constructed by ANI are more consistent with the genome‐ and protein‐based phylogenetic trees published previously than the 16S rRNA tree (Bonacina et al., 2016). The function of the *E. hirae* R17‐specific proteins suggested that this isolate may have unique carbohydrate utilization processes and a cell wall/membrane/envelope constitution. In a recent report, the overall genomes of six pig fecal *E. hirae* isolates were found to be highly similar to each other and to the type strain *E. hirae* ATCC^™^9790, but the gene content and structural differences are likely related to ecological niche specialization and may influence their competitiveness among strains (Katyal et al., 2016).

Genomic islands are known to contribute to the development of pathogenicity and the diversity that can be contained within a single bacterial species. Several studies have reported that the enterococci that cause infection in hospitalized patients are different from those isolates that colonize the gastrointestinal tract of the healthy human host, suggesting that the gain and loss of mobile genetic elements, rather than evolutionary descent, may be the most important driving force in determining virulence‐associated properties in isolates (Qin et al., 2012; Waters et al., 2011). In this study, two possible virulence factors, a toxin‐encoding gene and a hyaluronidase gene, located on GIs, strongly indicated that these pathogenesis‐related genes originated by HGT.

Capsular and other cell envelope polysaccharides of several Gram‐positive bacteria are known to play important roles in virulence and protective immunity (Thurlow, Thomas, Fleming, & Hancock, 2009a; Thurlow, Thomas, & Hancock, 2009b). Among the predicted virulence factors identified in the genome of *E. hirae* R17, some may contribute to pathogenesis and evasion of the host innate immune response (Jeon et al., 2015; Lee et al., 2015). Similar to other Gram‐positive bacteria, MSCRAMM (Microbial Surface Components Recognizing Adhesive Matrix Molecules)‐like cell wall‐anchored proteins were identified in *E. hirae* R17, including the biofilm‐associated Ebp pili, known to be important for endocarditis and UTI in animal models (Sillanpaa et al., 2010). The hyaluronidase function encoded by the *hyl* gene is primarily found in clinical *E. faecium* isolates and is much less common in isolates recovered from other sources (Rice et al., 2003). The conjugative transfer of a plasmid containing the *hyl* gene makes the transconjugant more virulent in a mouse peritonitis models, suggesting a role of hyaluronidase in virulence (Arias et al., 2009). The endocarditis enterococcal antigen (EfaA) is commonly found both among *E. faecalis* and *E. faecium*, and is presumed to be involved in the adhesion of enterococci to biotic and abiotic surfaces or evasion of the immune response (Abriouel et al., 2008). In addition, the binding to extracellular matrix components of a number of other surface proteins may also contribute to the virulence of *E. hirae* R17 by acting as adhesions.

The clinical importance of the genus *Enterococcus* is directly related to its antibiotic resistance, which significantly contributes to the risk of colonization and infection. Relative to the streptococci, enterococci are intrinsically resistant to many commonly used antimicrobial agents, including most cephalosporins, all semisynthetic penicillins, and clindamycin. The intrinsic resistance to clindamycin is mediated by the gene product of *lsa*, which is also found in the *E. hirae* R17 genome, although its mechanism of action remains poorly defined (Bonacina et al., 2016; Hollenbeck & Rice, 2012). Enterococcal isolates are often tolerant to β‐lactam antibiotics, likely due to the expression of low‐affinity penicillin‐binding proteins (PBPs) (Arias & Murray, 2012). Although four genes encoding PBPs (denoted as PBP2b, PBP1b, PBP1a, and PBP2x) were identified in the *E. hirae* R17 chromosome, this bacterium is susceptible to penicillin and ampicillin.

In addition to intrinsic resistance, enterococci have been extraordinarily successful at exploiting mechanisms of gene mobility and exchange to rapidly acquire resistance to virtually any antimicrobial agent in current use (Munita et al., 2015). The regular use of antimicrobials agents in modern animal production potentially increases selection pressure on enterococci to become resistant for niche adaptation and also increases the risk of drug‐resistant genes spreading from those bacteria in food‐producing animals to humans (Bonacina et al., 2016; Koluman & Dikici, 2013; Peng et al., 2014; Van Boeckel et al., 2015). Analysis of the drug‐resistance genes identified in *E. hirae* R17 suggests that this bacterium acquires these features by horizontal means involving plasmids, transposons, and insertion sequences. As an example, a 15 kb resistance gene island in plasmid pRZ1 was found to be composed of four IS*6* elements, which may function to disseminate genes to neighboring bacteria. This cluster contained genes encoding resistance to bacitracin (*bcrD*,* bcrB*,* bcrA*, and *bcrR*), macrolides (*ermB*), and tetracycline (*tetK* and *tetT*).

Bacitracin is used both in clinical practice and as an animal growth promoter in some countries. There were recent reports of high‐level bacitracin resistance in the genus *Enterococcus* (Kelesidis, 2015). The *bcr* operon is often clustered in one region on the enterococcal genome; however, it remains unclear why there is one orphan *bcrD* gene on the chromosome of *E. hirae* R17 and a complete *bcr* operon on the plasmid.

DAP is a lipopeptide antibiotic frequently used as a last‐resort antibiotic against vancomycin‐resistant enterococci. There are limited treatment options for DAP‐resistant enterococcal isolates (Tang et al., 2015). Other than *E. faecalis* and *E. faecium*, this is the first report of DAP‐resistant bacterium in *E. hirae*. The genetic basis and supporting mechanism for this DAP resistance of enterococci is complex and poorly understood. The changes in regulatory systems that control the cell envelope response to stress may be critical to the development of DAP resistance in enterococci clinical isolates (Diaz et al., 2014). In the chromosome of *E. hirae* R17, the DAP resistance‐associated genes *mprF* and *cls* were found, and these may contribute to the observed DAP resistance of *E. hirae* R17 (Arias et al., 2012). Further studies on the mechanisms of DAP resistance in *E. hirae* R17 are warranted, because DAP is not typically used in animal production in China due to its high price.

Antibacterial biocides and metals can contribute to the development and maintenance of antibiotic resistance in bacterial communities through co‐selection (Dortet, Anguel, Fortineau, Richard, & Nordmann, 2013; Pal et al., 2014). Based on the analysis of the genome of *E. hirae* R17, genes encoding resistance to several heavy metals, including copper, iron, silver, and also selenium, were identified. This finding is consistent with the origin of *E. hirae* R17, given the fact that copper, selenium, and iron are often used as growth‐promoting pig feed supplements. The potential resistance of *E. hirae* R17 to hydrochloric acid, hydrogen peroxide, and sodium dodecyl sulfate indicated that this bacterium exhibits a robust ability to withstand adverse environment. Because the tetracycline resistance gene *tet*(K) is also an antibacterial biocide gene (*lmrS*), it is possible that the resistance of *E. hirae* R17 to tetracycline could be transferred to surrounding bacteria under the pressure of an antibacterial biocide.

IS elements and transposable elements are short DNA sequences capable of independent transposition within and between bacterial genomes. Evolution experiments demonstrate that these mobile genetic elements contribute to the generation of genetic diversity and can help promote adaptation of the host bacteria (Zhang et al., 2014). The ability of enterococci to acquire mobile genetic elements encoding traits has contributed to the emergence of these bacteria as leading hospital pathogens. Antibiotic resistance and virulence traits have accumulated in lineages associated with hospital infection outbreaks, such as the *E. faecium* clonal complex CC 17 (Waters et al., 2011) and *E. faecalis* CC2 (Anderson et al., 2015). In addition, bacteria can exploit the instability of TRs to reversibly shut down or modulate the function of specific genes, allowing adaptation to changing environments on short evolutionary time scales without an increased overall mutation rate (Munita et al., 2015). Therefore, the presence of IS sequences and several TRs indicate that *E. hirae* R17 has strong genomic plasticity with potential for environmental adaption. The results of this study also suggest that the R17 plasmid may be responsible for much of the horizontal gene transfer that has allowed antibiotic, virulence, and antibacterial biocide traits to converge in clinical adapted lineages.

Mobile elements may provide an accessory pool of adaptive genes that, if acquired by a bacterium, may have the potential to enhance survival in select environments. Paradoxically, the CRISPR‐*cas* system in bacteria confers resistance against foreign genetic elements, providing adaptive immunity against foreign DNA including plasmids and bacteriophage (Chuang et al., 2014). One hypothesis suggests that an inverse relationship exists between the acquisition of one or more antimicrobial resistance‐encoding genes and the possession of complete CRISPR loci. This appears to hold for members of the recently emerged high‐risk *E. faecium* and *E. faecalis* lineages, as such bacteria lack these loci. Surprisingly, this hypothesis does not appear to hold in the case of *E. hirae* R17, as a CRISPR1‐*cas* loci was identified (Table 1). The incorporation of these components may have been stimulated by modern antibiotic therapy, promoting the acquisition of foreign elements conferring antibiotic resistance, decreasing genome stability/increasing plasticity, and enabling the colonization of new habitats, including the antibiotic‐laden hospital environment (O'Driscoll & Crank, 2015).

It has been suggested previously that the pan‐genome of *E. faecium* is essentially unlimited in size. This may indicate that *E. faecium* has the potential to acquire and incorporate exogenous DNA into its genome (van Schaik et al., 2010). In contrast, our previously limited genomic information of *E. hirae* has hindered the prediction of core and pan genomic information of this species. Additionally, there is a need for a multistrain genomic analysis of *E. hirae* to describe its fundamental biology and to determine the causes of its emergence as a possible opportunistic pathogen.

## 5 CONCLUSION

The *E. hirae* R17 genome sequence provides a starting point to study the evolutionary history and the potential pathobiology of this species, particularly those isolates recovered from food‐related samples. We found that the isolate originating from retailed pork acquired multiple antibiotic resistance genes and also has genes that may play roles in colonization and the infection of animal food consumers. The genomic flexibility of *E. hirae* R17, with its IS elements, transposase, and TRs, allows rapid adaptation to new environmental niches such as those that are found in food sterilization and storage sites. Genome‐wide studies, which will be facilitated by the sequence data presented here, are therefore needed to increase our understanding of the basic biology of *E. hirae* and to identify genes that may present risks for public health.

## CONFLICT OF INTEREST

The authors declare that they have no conflict of interests.
