# Supplementary material for: Genomic insights into the pathogenicity and environmental adaptability of Enterococcus hirae R17 isolated from pork offered for retail sale
Source: Microbiologyopen. 2017 Aug 10;6(6):e00514. doi: 10.1002/mbo3.514 (PMC5727370; doi:10.1002/mbo3.514)
Supplement: Supplementary file 1 [file MBO3-6-na-s001.doc]

**Supplementary Table S1.** Strain-specific genes in *E. hirae* R17 in comparison with *E. hirae* ATCC™9790

| Specific region | Gene locus_tag | Protein ID | Annotation function | COG category* |
| --- | --- | --- | --- | --- |
|  | A6P53_00020 | AND71320.1 | DNA replication/repair protein RecF | L |
|  | A6P53_00140 | AND71342.1 | methyltransferase | J |
|  | A6P53_00270 | AND71367.1 | fructose-bisphosphate aldolase | G |
|  | A6P53_00450 | AND71401.1 | hypothetical protein | none |
|  | A6P53_00455 | AND71402.1 | hypothetical protein | none |
|  | A6P53_00625 | AND71434.1 | hypothetical protein | none |
|  | A6P53_00670 | AND71443.1 | hypothetical protein | none |
|  | A6P53_00680 | AND71444.1 | hypothetical protein | none |
|  | A6P53_00685 | AND71445.1 | hypothetical protein | none |
|  | A6P53_00705 | AND71448.1 | 6-O-methylguanine DNA methyltransferase | L |
|  | A6P53_00825 | AND71471.1 | hypothetical protein | none |
|  | A6P53_00830 | AND71472.1 | hypothetical protein | none |
| Region 1^&^ | A6P53_01005 | AND71504.1 | hypothetical protein | none |
|  | A6P53_01010 | AND71505.1 | hypothetical protein | none |
|  | A6P53_01015 | AND71506.1 | UDP-N-acetyl-D-mannosamine transferase | M |
|  | A6P53_01020 | AND71507.1 | hypothetical protein | M |
|  | A6P53_01025 | AND71508.1 | hypothetical protein | none |
|  | A6P53_01030 | AND71509.1 | GlcNAc transferase | M |
|  | A6P53_01035 | AND71510.1 | galactoside O-acetyltransferase | R |
|  | A6P53_01040 | AND71511.1 | hypothetical protein | none |
|  | A6P53_01045 | AND71512.1 | hypothetical protein | none |
|  | A6P53_01050 | AND71513.1 | hypothetical protein | C |
|  | A6P53_01090 | AND71521.1 | hypothetical protein | none |
|  | A6P53_01170 | AND71536.1 | hypothetical protein | none |
|  | A6P53_01200 | AND71541.1 | hypothetical protein | none |
|  | A6P53_01205 | AND71542.1 | hypothetical protein | none |
|  | A6P53_01400 | AND71577.1 | LacI family transcriptional regulator | K |
|  | A6P53_01530 | AND71599.1 | M protein trans-acting positive regulator | none |
|  | A6P53_01550 | AND71603.1 | class C sortase | M |
|  | A6P53_01575 | AND71607.1 | 4-hydroxy-tetrahydrodipicolinate reductase | E |
|  | A6P53_01610 | AND71614.1 | DNA-binding protein | K |
|  | A6P53_01705 | AND71633.1 | hypothetical protein | none |
|  | A6P53_01800 | AND71652.1 | hypothetical protein | none |
|  | A6P53_01805 | AND71653.1 | hypothetical protein | none |
|  | A6P53_01810 | AND71654.1 | hypothetical protein | none |
|  | A6P53_02035 | AND71695.1 | fatty acid-binding protein DegV | none |
|  | A6P53_02065 | AND71701.1 | hypothetical protein | none |
|  | A6P53_02080 | AND71704.1 | hypothetical protein | none |
|  | A6P53_02110 | AND71710.1 | LacI family transcriptional regulator | K |
|  | A6P53_02165 | AND71719.1 | AI-2E family transporter | R |
|  | A6P53_02425 | AND71766.1 | methionine ABC transporter permease | P |
|  | A6P53_02470 | AND71775.1 | TIGR04141 family sporadically distributed protein | none |
|  | A6P53_02480 | AND71776.1 | hypothetical protein | none |
|  | A6P53_02485 | AND71777.1 | arsenate reductase (thioredoxin) | T |
|  | A6P53_02490 | AND71778.1 | hypothetical protein | none |
|  | A6P53_02510 | AND71781.1 | hypothetical protein | none |
|  | A6P53_02570 | AND71792.1 | helix-turn-helix transcriptional regulator | K |
|  | A6P53_02575 | AND71793.1 | hypothetical protein | none |
|  | A6P53_02595 | AND71797.1 | hypothetical protein | K |
|  | A6P53_02685 | AND71813.1 | L-seryl-tRNA selenium transferase | E |
|  | A6P53_02830 | AND71841.1 | oligosaccharide biosynthesis protein Alg14 | none |
|  | A6P53_03120 | AND71898.1 | two-component sensor histidine kinase | T |
|  | A6P53_03155 | AND71905.1 | DNA polymerase IV | L |
|  | A6P53_03175 | AND71909.1 | ABC transporter permease | V |
|  | A6P53_03440 | AND71960.1 | RNA-binding protein | S |
|  | A6P53_03460 | AND71964.1 | hypothetical protein | none |
|  | A6P53_03495 | AND71971.1 | RNA helicase | LKJ |
|  | A6P53_03780 | AND72026.1 | hypothetical protein | none |
|  | A6P53_03790 | AND72028.1 | hypothetical protein | none |
|  | A6P53_03925 | AND72054.1 | 3-oxoacyl-ACP synthase | I |
|  | A6P53_03960 | AND72060.1 | acetyl-CoA carboxylase biotin carboxylase subunit | I |
|  | A6P53_04240 | AND72096.1 | transcriptional regulator | S |
|  | A6P53_04355 | AND72117.1 | hypothetical protein | none |
|  | A6P53_04730 | AND72186.1 | thymidylate synthase | F |
|  | A6P53_04795 | AND72199.1 | ribonuclease HII | L |
|  | A6P53_04940 | AND72228.1 | hypothetical protein | none |
|  | A6P53_05085 | AND72256.1 | xylulose 5-phosphate 3-epimerase | G |
|  | A6P53_05225 | AND72283.1 | "inosine monophosphate cyclohydrolase"" |  |
|  | " | F |  |  |
|  | A6P53_05275 | AND72293.1 | hypothetical protein | none |
| Region 2 | A6P53_05285 | AND72294.1 | hypothetical protein | none |
|  | A6P53_05290 | AND72295.1 | hypothetical protein | none |
|  | A6P53_05295 | AND72296.1 | hypothetical protein | none |
|  | A6P53_05300 | AND72297.1 | hypothetical protein | none |
|  | A6P53_05305 | AND72298.1 | hypothetical protein | none |
|  | A6P53_05310 | AND72299.1 | hypothetical protein | none |
|  | A6P53_05315 | AND72300.1 | hypothetical protein | S |
|  | A6P53_05465 | AND72329.1 | hypothetical protein | none |
|  | A6P53_05475 | AND72331.1 | hypothetical protein | S |
|  | A6P53_05575 | AND72349.1 | phenylalanine--tRNA ligase subunit alpha | J |
|  | A6P53_05610 | AND72356.1 | hypothetical protein | none |
|  | A6P53_05630 | AND72357.1 | hypothetical protein | none |
|  | A6P53_05655 | AND72362.1 | hypothetical protein | none |
|  | A6P53_05660 | AND72363.1 | hypothetical protein | none |
|  | A6P53_05690 | AND72369.1 | mechanosensitive ion channel protein MscS | M |
|  | A6P53_05795 | AND72389.1 | cysteine synthase A | E |
|  | A6P53_05895 | AND72408.1 | DEAD/DEAH box helicase | LKJ |
|  | A6P53_05935 | AND72415.1 | hypothetical protein | none |
|  | A6P53_05960 | AND72418.1 | hypothetical protein | none |
|  | A6P53_06105 | AND72447.1 | Cro/Cl family transcriptional regulator | K |
|  | A6P53_06110 | AND72448.1 | hypothetical protein | none |
|  | A6P53_06135 | AND72453.1 | hypothetical protein | none |
|  | A6P53_06140 | AND72454.1 | hypothetical protein | none |
|  | A6P53_06145 | AND72455.1 | hypothetical protein | none |
|  | A6P53_06160 | AND72458.1 | hypothetical protein | none |
|  | A6P53_06165 | AND72459.1 | hypothetical protein | none |
|  | A6P53_06180 | AND72462.1 | hypothetical protein | none |
|  | A6P53_06220 | AND72469.1 | integrase | L |
|  | A6P53_06275 | AND72480.1 | branched-chain amino acid ABC transporter | E |
|  | A6P53_06290 | AND72483.1 | viral A-type inclusion protein | none |
|  | A6P53_06305 | AND72486.1 | pyrimidine nucleoside transporter NupC | F |
|  | A6P53_06315 | AND72488.1 | phage tail protein | none |
|  | A6P53_06405 | AND72506.1 | proline reductase | none |
|  | A6P53_06440 | AND72513.1 | hypothetical protein | none |
|  | A6P53_06450 | AND72515.1 | maltodextrose utilization protein MalA | S |
|  | A6P53_06480 | AND72520.1 | 2-succinyl-6-hydroxy-2,4-cyclohexadiene-1-carboxylate synthase | R |
|  | A6P53_06540 | AND72532.1 | M protein trans-acting positive regulator | none |
|  | A6P53_06720 | AND72567.1 | isochorismatase | Q |
|  | A6P53_06760 | AND72574.1 | hypothetical protein | none |
|  | A6P53_06765 | AND72575.1 | cell surface protein | none |
|  | A6P53_06770 | AND72576.1 | cell surface protein | none |
|  | A6P53_06775 | AND72577.1 | DNA-binding protein | none |
|  | A6P53_06820 | AND72584.1 | hypothetical protein | none |
| Region 3 | A6P53_06915 | AND72601.1 | hypothetical protein | none |
|  | A6P53_06920 | AND72602.1 | hypothetical protein | none |
|  | A6P53_06925 | AND72603.1 | hypothetical protein | none |
|  | A6P53_06930 | AND72604.1 | hypothetical protein | none |
|  | A6P53_06935 | AND72605.1 | hypothetical protein | none |
|  | A6P53_06940 | AND72606.1 | antirestriction protein ArdA | R |
|  | A6P53_06945 | AND72607.1 | transcriptional regulator | K |
|  | A6P53_06950 | AND72608.1 | hypothetical protein | none |
|  | A6P53_06955 | AND73702.1 | hypothetical protein | none |
|  | A6P53_06960 | AND72609.1 | hypothetical protein | none |
|  | A6P53_07005 | AND72618.1 | SprT family protein | S |
|  | A6P53_07020 | AND72621.1 | hypothetical protein | none |
|  | A6P53_07110 | AND72637.1 | PTS glucose transporter subunit IIABC | G |
|  | A6P53_07145 | AND72646.1 | histidine kinase | T |
|  | A6P53_07185 | AND72654.1 | oxidoreductase | none |
|  | A6P53_07340 | AND72683.1 | hypothetical protein | S |
|  | A6P53_07350 | AND72685.1 | pyruvate:ferredoxin (flavodoxin) oxidoreductase | C |
|  | A6P53_07440 | AND73704.1 | hypothetical protein | R |
|  | A6P53_07445 | AND72702.1 | addiction module antitoxin | none |
|  | A6P53_07450 | AND72703.1 | hypothetical protein | none |
|  | A6P53_07460 | AND73705.1 | hypothetical protein | none |
|  | A6P53_07475 | AND72705.1 | restriction endonuclease | V |
|  | A6P53_07505 | AND72709.1 | transcriptional regulator | K |
|  | A6P53_07525 | AND72712.1 | beta-glucosidase | G |
|  | A6P53_07540 | AND72715.1 | sugar ABC transporter permease | G |
|  | A6P53_07610 | AND73707.1 | radical SAM/SPASM domain-containing protein | R |
|  | A6P53_07630 | AND72732.1 | tRNA pseudouridine(55) synthase | J |
|  | A6P53_07755 | AND72756.1 | glycosyltransferase | M |
|  | A6P53_07770 | AND73709.1 | tyrosine protein kinase | D |
|  | A6P53_07805 | AND72764.1 | ribosomal-protein-alanine N-acetyltransferase RimI | R |
|  | A6P53_07920 | AND72786.1 | hypothetical protein | none |
|  | A6P53_07930 | AND72788.1 | hypothetical protein | K |
|  | A6P53_07940 | AND72789.1 | hypothetical protein | none |
|  | A6P53_07945 | AND72790.1 | hypothetical protein | none |
|  | A6P53_07950 | AND72791.1 | peptidoglycan-binding protein LysM | none |
|  | A6P53_07975 | AND72795.1 | hypothetical protein | none |
|  | A6P53_07995 | AND72799.1 | glucosyltransferase | none |
|  | A6P53_08015 | AND72802.1 | hypothetical protein | none |
|  | A6P53_08020 | AND72803.1 | hypothetical protein | none |
|  | A6P53_08050 | AND72809.1 | hypothetical protein | none |
| Region 4 | A6P53_08065 | AND72810.1 | hypothetical protein | SM |
|  | A6P53_08070 | AND72811.1 | phage tail protein | S |
|  | A6P53_08080 | AND72812.1 | hypothetical protein | none |
|  | A6P53_08085 | AND72813.1 | phage tail protein | none |
|  | A6P53_08090 | AND72814.1 | hypothetical protein | none |
|  | A6P53_08100 | AND72815.1 | hypothetical protein | none |
|  | A6P53_08105 | AND72816.1 | hypothetical protein | none |
|  | A6P53_08110 | AND72817.1 | hypothetical protein | none |
|  | A6P53_08115 | AND72818.1 | hypothetical protein | none |
|  | A6P53_08120 | AND72819.1 | hypothetical protein | none |
|  | A6P53_08125 | AND72820.1 | hypothetical protein | none |
|  | A6P53_08130 | AND73714.1 | phage head morphogenesis protein | none |
|  | A6P53_08145 | AND72821.1 | small subunit of terminase | S |
|  | A6P53_08155 | AND72823.1 | hypothetical protein | none |
|  | A6P53_08160 | AND72824.1 | hypothetical protein | none |
|  | A6P53_08170 | AND72826.1 | hypothetical protein | none |
|  | A6P53_08195 | AND72831.1 | hypothetical protein | none |
|  | A6P53_08200 | AND72832.1 | hypothetical protein | none |
|  | A6P53_08220 | AND72836.1 | hypothetical protein | none |
|  | A6P53_08225 | AND72837.1 | DNA-binding protein | none |
|  | A6P53_08230 | AND72838.1 | hypothetical protein | none |
|  | A6P53_08245 | AND72841.1 | transcriptional regulator | none |
|  | A6P53_08260 | AND72844.1 | hypothetical protein | none |
|  | A6P53_08375 | AND72865.1 | hypothetical protein | none |
|  | A6P53_08385 | AND72866.1 | hypothetical protein | S |
|  | A6P53_08390 | AND72867.1 | hypothetical protein | none |
|  | A6P53_08400 | AND72868.1 | hypothetical protein | none |
|  | A6P53_08415 | AND72869.1 | hypothetical protein | none |
|  | A6P53_08420 | AND72870.1 | Pin-related site-specific recombinase/DNA invertase | L |
|  | A6P53_08495 | AND72883.1 | Xaa-Pro aminopeptidase | none |
|  | A6P53_08500 | AND72884.1 | DeoR family transcriptional regulator | KG |
| Region 5 | A6P53_08520 | AND72887.1 | potassium transporter KtrB | P |
|  | A6P53_08525 | AND72888.1 | potassium-transporting ATPase subunit B | P |
|  | A6P53_08530 | AND72889.1 | potassium-transporting ATPase subunit KdpA | P |
|  | A6P53_08535 | AND72890.1 | tagatose-bisphosphate aldolase | G |
|  | A6P53_08540 | AND72891.1 | galactose-6-phosphate isomerase | G |
|  | A6P53_08545 | AND72892.1 | galactose-6-phosphate isomerase | G |
|  | A6P53_08550 | AND72893.1 | 6-phospho-beta-galactosidase | G |
|  | A6P53_08555 | AND72894.1 | PTS lactose transporter subunit IIBC | G |
|  | A6P53_08560 | AND72895.1 | PTS lactose transporter subunit IIA | G |
|  | A6P53_08565 | AND72896.1 | DeoR family transcriptional regulator | KG |
|  | A6P53_08570 | AND72897.1 | transketolase | G |
|  | A6P53_08575 | AND72898.1 | hypothetical protein | G |
|  | A6P53_08700 | AND72922.1 | hypothetical protein | none |
|  | A6P53_08965 | AND72972.1 | hypothetical protein | none |
|  | A6P53_08990 | AND72977.1 | multidrug ABC transporter ATP-binding protein | V |
|  | A6P53_09085 | AND72996.1 | hypothetical protein | none |
|  | A6P53_09165 | AND73011.1 | hypothetical protein | none |
|  | A6P53_09190 | AND73014.1 | hypothetical protein | none |
|  | A6P53_09365 | AND73044.1 | glycine/betaine/sarcosine/D-proline reductase family selenoprotein B | none |
|  | A6P53_09400 | AND73050.1 | hypothetical protein | none |
|  | A6P53_09410 | AND73051.1 | hypothetical protein | none |
|  | A6P53_09435 | AND73056.1 | lipoate--protein ligase | H |
|  | A6P53_09525 | AND73073.1 | excinuclease ABC subunit C | L |
|  | A6P53_09555 | AND73079.1 | chitinase | R |
|  | A6P53_09565 | AND73081.1 | hypothetical protein | none |
|  | A6P53_09575 | AND73083.1 | hemin ABC transporter ATP-binding protein | V |
|  | A6P53_09610 | AND73090.1 | hypothetical protein | none |
| Region 6 | A6P53_09640 | AND73095.1 | hypothetical protein | K |
|  | A6P53_09645 | AND73096.1 | multidrug ABC transporter ATP-binding protein | V |
|  | A6P53_09650 | AND73097.1 | ABC transporter | V |
|  | A6P53_09655 | AND73098.1 | hypothetical protein | none |
|  | A6P53_09660 | AND73099.1 | hypothetical protein | none |
|  | A6P53_09850 | AND73135.1 | hypothetical protein | none |
|  | A6P53_09985 | AND73160.1 | septation ring formation regulator EzrA | D |
|  | A6P53_10105 | AND73178.1 | multifunctional 2',3'-cyclic-nucleotide 2'-phosphodiesterase/5'-nucleotidase/3'-nucleotidase | F |
|  | A6P53_10145 | AND73727.1 | ribonuclease P protein component | none |
|  | A6P53_10350 | AND73223.1 | transposase | L |
| Region 7 | A6P53_10365 | AND73226.1 | hypothetical protein | none |
|  | A6P53_10370 | AND73227.1 | hypothetical protein | none |
|  | A6P53_10375 | AND73228.1 | hypothetical protein | M |
|  | A6P53_10380 | AND73229.1 | hypothetical protein | none |
|  | A6P53_10385 | AND73230.1 | hypothetical protein | none |
|  | A6P53_10395 | AND73232.1 | hypothetical protein | none |
|  | A6P53_10400 | AND73233.1 | hypothetical protein | none |
|  | A6P53_10405 | AND73234.1 | hypothetical protein | T |
|  | A6P53_10410 | AND73235.1 | hypothetical protein | L |
|  | A6P53_10415 | AND73236.1 | hypothetical protein | none |
|  | A6P53_10420 | AND73237.1 | transposase | L |
|  | A6P53_10425 | AND73238.1 | hypothetical protein | none |
|  | A6P53_10435 | AND73240.1 | UDP-N-acetyl-D-glucosamine dehydrogenase | M |
|  | A6P53_10440 | AND73241.1 | hypothetical protein | M |
|  | A6P53_10450 | AND73243.1 | hypothetical protein | none |
| Region 8 | A6P53_10460 | AND73245.1 | hypothetical protein | none |
|  | A6P53_10465 | AND73246.1 | hypothetical protein | GM |
|  | A6P53_10470 | AND73247.1 | group 1 glycosyl transferase | none |
|  | A6P53_10475 | AND73248.1 | hypothetical protein | S |
|  | A6P53_10480 | AND73249.1 | hypothetical protein | none |
|  | A6P53_10485 | AND73250.1 | glycosyl transferase | M |
|  | A6P53_10490 | AND73251.1 | hypothetical protein | M |
|  | A6P53_10495 | AND73252.1 | UDP-N-acetyl-D-mannosamine dehydrogenase | M |
|  | A6P53_10500 | AND73253.1 | hypothetical protein | none |
|  | A6P53_10560 | AND73265.1 | amino acid ABC transporter substrate-binding protein |  |
|  | " | ET |  |  |
|  | A6P53_10625 | AND73276.1 | hypothetical protein | none |
|  | A6P53_10970 | AND73345.1 | ribosomal protein L11 methyltransferase | J |
|  | A6P53_11190 | AND73382.1 | sodium:dicarboxylate symporter | C |
|  | A6P53_11195 | AND73383.1 | MFS transporter | GEPR |
|  | A6P53_11200 | AND73732.1 | hypothetical protein | none |
|  | A6P53_11205 | AND73731.1 | hypothetical protein | C |
|  | A6P53_11215 | AND73384.1 | transcriptional regulator | TQ |
|  | A6P53_11260 | AND73393.1 | hypothetical protein | none |
|  | A6P53_11280 | AND73733.1 | guanosine 5'-monophosphate oxidoreductase | V |
|  | A6P53_11290 | AND73734.1 | deoxyribonuclease | V |
|  | A6P53_11310 | AND73398.1 | bacteriochlorophyll 4-vinyl reductase | none |
| Region 9 | A6P53_11330 | AND73402.1 | glucose-6-phosphate isomerase | GR |
|  | A6P53_11335 | AND73403.1 | mannose-6-phosphate isomerase | G |
|  | A6P53_11340 | AND73404.1 | hypothetical protein | G |
|  | A6P53_11345 | AND73405.1 | PTS fructose transporter subunit IID | G |
|  | A6P53_11350 | AND73406.1 | PTS sorbose transporter subunit IIC | G |
|  | A6P53_11355 | AND73407.1 | PTS system sorbose subfamily IIB component | G |
|  | A6P53_11360 | AND73408.1 | GntR family transcriptional regulator | K |
|  | A6P53_11405 | AND73417.1 | glycosyl transferase | M |
|  | A6P53_11475 | AND73735.1 | hypothetical protein | none |
|  | A6P53_11550 | AND73445.1 | MarR family transcriptional regulator | K |
|  | A6P53_11665 | AND73467.1 | cyclic nucleotide-binding protein | S |
|  | A6P53_11675 | AND73737.1 | cell wall hydrolase | none |
|  | A6P53_12140 | AND73530.1 | primosomal protein N | L |
|  | A6P53_12245 | AND73551.1 | competence/damage-inducible protein A | R |
|  | A6P53_12345 | AND73571.1 | hypothetical protein | none |
|  | A6P53_12350 | AND73572.1 | hypothetical protein | none |
|  | A6P53_12355 | AND73573.1 | MFS transporter | GEPR |
|  | A6P53_12435 | AND73588.1 | DNA mismatch repair protein MutS | L |
|  | A6P53_12510 | AND73601.1 | type III restriction endonuclease subunit R | KL |
|  | A6P53_12515 | AND73602.1 | adenine methyltransferase | V |
|  | A6P53_12525 | AND73603.1 | hypothetical protein | none |
|  | A6P53_12800 | AND73746.1 | beta-ketoacyl-[acyl-carrier-protein] synthase II | IQ |

^*^ reprents functional categories of strain-specific genes of *E. hirae* R17. C, Energy production and conversion; D, Cell cycle control, cell division, chromosome partitioning; E, Amino acid transport and metabolism; F, Nucleotide transport and metabolism; G, Carbohydrate transport and metabolism; H, Coenzyme transport and metabolism; I, Lipid transport and metabolism; J, Translation, ribosomal structure and biogenesis; K, Transcription; L, Replication, recombination and repair; M, Cell wall/membrane/envelope biogenesis; P, Inorganic ion transport and metabolism; Q, Secondary metabolites biosynthesis, transport and catabolism; R, General function prediction only; S, Function unknown; T, Signal transduction mechanisms; V, Defense mechanisms; none, no functional category.

^&^ Region contains more than five consecutive strain-specific genes.

**Supplementary Table S2.** Strain-specific genes in *E. hirae* R17 in comparison with *E. hirae* ATCC™9790, *E. faecium* DO, and *E. faecalis* V583.

| Specific region | Gene locus_tag | Protein ID | Annotation function | COG category |
| --- | --- | --- | --- | --- |
|  | A6P53_00270 | AND71367.1 | fructose-bisphosphate aldolase | G |
|  | A6P53_00625 | AND71434.1 | hypothetical protein | none |
|  | A6P53_00670 | AND71443.1 | hypothetical protein | none |
|  | A6P53_00680 | AND71444.1 | hypothetical protein | none |
|  | A6P53_00685 | AND71445.1 | hypothetical protein | none |
| Region #1* | A6P53_01020 | AND71507.1 | hypothetical protein | M |
|  | A6P53_01025 | AND71508.1 | hypothetical protein | none |
|  | A6P53_01030 | AND71509.1 | GlcNAc transferase | M |
|  | A6P53_01035 | AND71510.1 | galactoside O-acetyltransferase | R |
|  | A6P53_01040 | AND71511.1 | hypothetical protein | none |
|  | A6P53_01045 | AND71512.1 | hypothetical protein | none |
|  | A6P53_01050 | AND71513.1 | hypothetical protein | C |
|  | A6P53_01090 | AND71521.1 | hypothetical protein | none |
|  | A6P53_01170 | AND71536.1 | hypothetical protein | none |
|  | A6P53_01200 | AND71541.1 | hypothetical protein | none |
|  | A6P53_01205 | AND71542.1 | hypothetical protein | none |
|  | A6P53_01530 | AND71599.1 | M protein trans-acting positive regulator | none |
|  | A6P53_01610 | AND71614.1 | DNA-binding protein | K |
|  | A6P53_01705 | AND71633.1 | hypothetical protein | none |
|  | A6P53_01810 | AND71654.1 | hypothetical protein | none |
|  | A6P53_02080 | AND71704.1 | hypothetical protein | none |
|  | A6P53_02470 | AND71775.1 | TIGR04141 family sporadically distributed protein | none |
|  | A6P53_02480 | AND71776.1 | hypothetical protein | none |
|  | A6P53_02490 | AND71778.1 | hypothetical protein | none |
|  | A6P53_02510 | AND71781.1 | hypothetical protein | none |
|  | A6P53_02575 | AND71793.1 | hypothetical protein | none |
|  | A6P53_02595 | AND71797.1 | hypothetical protein | K |
|  | A6P53_03460 | AND71964.1 | hypothetical protein | none |
|  | A6P53_03790 | AND72028.1 | hypothetical protein | none |
|  | A6P53_04355 | AND72117.1 | hypothetical protein | none |
|  | A6P53_05275 | AND72293.1 | hypothetical protein | none |
|  | A6P53_05290 | AND72295.1 | hypothetical protein | none |
|  | A6P53_05295 | AND72296.1 | hypothetical protein | none |
|  | A6P53_05310 | AND72299.1 | hypothetical protein | none |
|  | A6P53_05465 | AND72329.1 | hypothetical protein | none |
|  | A6P53_05475 | AND72331.1 | hypothetical protein | S |
|  | A6P53_05610 | AND72356.1 | hypothetical protein | none |
|  | A6P53_05630 | AND72357.1 | hypothetical protein | none |
|  | A6P53_05655 | AND72362.1 | hypothetical protein | none |
|  | A6P53_05660 | AND72363.1 | hypothetical protein | none |
|  | A6P53_05935 | AND72415.1 | hypothetical protein | none |
|  | A6P53_06110 | AND72448.1 | hypothetical protein | none |
|  | A6P53_06135 | AND72453.1 | hypothetical protein | none |
|  | A6P53_06140 | AND72454.1 | hypothetical protein | none |
|  | A6P53_06145 | AND72455.1 | hypothetical protein | none |
|  | A6P53_06160 | AND72458.1 | hypothetical protein | none |
|  | A6P53_06165 | AND72459.1 | hypothetical protein | none |
|  | A6P53_06180 | AND72462.1 | hypothetical protein | none |
|  | A6P53_06405 | AND72506.1 | proline reductase | none |
|  | A6P53_06440 | AND72513.1 | hypothetical protein | none |
|  | A6P53_06760 | AND72574.1 | hypothetical protein | none |
|  | A6P53_06765 | AND72575.1 | cell surface protein | none |
|  | A6P53_06770 | AND72576.1 | cell surface protein | none |
|  | A6P53_06820 | AND72584.1 | hypothetical protein | none |
| Region #2 | A6P53_06915 | AND72601.1 | hypothetical protein | none |
|  | A6P53_06920 | AND72602.1 | hypothetical protein | none |
|  | A6P53_06925 | AND72603.1 | hypothetical protein | none |
|  | A6P53_06930 | AND72604.1 | hypothetical protein | none |
|  | A6P53_06935 | AND72605.1 | hypothetical protein | none |
|  | A6P53_06945 | AND72607.1 | transcriptional regulator | K |
|  | A6P53_06950 | AND72608.1 | hypothetical protein | none |
|  | A6P53_06955 | AND73702.1 | hypothetical protein | none |
|  | A6P53_06960 | AND72609.1 | hypothetical protein | none |
|  | A6P53_07020 | AND72621.1 | hypothetical protein | none |
|  | A6P53_07460 | AND73705.1 | hypothetical protein | none |
|  | A6P53_07475 | AND72705.1 | restriction endonuclease | V |
|  | A6P53_07505 | AND72709.1 | transcriptional regulator | K |
|  | A6P53_07525 | AND72712.1 | beta-glucosidase | G |
|  | A6P53_07540 | AND72715.1 | sugar ABC transporter permease | G |
|  | A6P53_07610 | AND73707.1 | radical SAM/SPASM domain-containing protein | R |
|  | A6P53_07770 | AND73709.1 | tyrosine protein kinase | D |
|  | A6P53_07920 | AND72786.1 | hypothetical protein | none |
|  | A6P53_07930 | AND72788.1 | hypothetical protein | K |
|  | A6P53_07945 | AND72790.1 | hypothetical protein | none |
|  | A6P53_08015 | AND72802.1 | hypothetical protein | none |
|  | A6P53_08020 | AND72803.1 | hypothetical protein | none |
|  | A6P53_08050 | AND72809.1 | hypothetical protein | none |
|  | A6P53_08105 | AND72816.1 | hypothetical protein | none |
|  | A6P53_08145 | AND72821.1 | small subunit of terminase | S |
|  | A6P53_08155 | AND72823.1 | hypothetical protein | none |
|  | A6P53_08160 | AND72824.1 | hypothetical protein | none |
|  | A6P53_08170 | AND72826.1 | hypothetical protein | none |
|  | A6P53_08220 | AND72836.1 | hypothetical protein | none |
|  | A6P53_08245 | AND72841.1 | transcriptional regulator | none |
|  | A6P53_08260 | AND72844.1 | hypothetical protein | none |
|  | A6P53_08375 | AND72865.1 | hypothetical protein | none |
|  | A6P53_08385 | AND72866.1 | hypothetical protein | S |
|  | A6P53_08390 | AND72867.1 | hypothetical protein | none |
|  | A6P53_08400 | AND72868.1 | hypothetical protein | none |
|  | A6P53_08415 | AND72869.1 | hypothetical protein | none |
|  | A6P53_08535 | AND72890.1 | tagatose-bisphosphate aldolase | G |
|  | A6P53_08570 | AND72897.1 | transketolase | G |
|  | A6P53_08575 | AND72898.1 | hypothetical protein | G |
|  | A6P53_08700 | AND72922.1 | hypothetical protein | none |
|  | A6P53_08965 | AND72972.1 | hypothetical protein | none |
|  | A6P53_09085 | AND72996.1 | hypothetical protein | none |
|  | A6P53_09400 | AND73050.1 | hypothetical protein | none |
|  | A6P53_09555 | AND73079.1 | chitinase | R |
|  | A6P53_09565 | AND73081.1 | hypothetical protein | none |
|  | A6P53_09610 | AND73090.1 | hypothetical protein | none |
| Region #3 | A6P53_09640 | AND73095.1 | hypothetical protein | K |
|  | A6P53_09645 | AND73096.1 | multidrug ABC transporter ATP-binding protein | V |
|  | A6P53_09650 | AND73097.1 | ABC transporter | V |
|  | A6P53_09655 | AND73098.1 | hypothetical protein | none |
|  | A6P53_09660 | AND73099.1 | hypothetical protein | none |
|  | A6P53_09850 | AND73135.1 | hypothetical protein | none |
| Region #4 | A6P53_10365 | AND73226.1 | hypothetical protein | none |
|  | A6P53_10370 | AND73227.1 | hypothetical protein | none |
|  | A6P53_10375 | AND73228.1 | hypothetical protein | M |
|  | A6P53_10380 | AND73229.1 | hypothetical protein | none |
|  | A6P53_10385 | AND73230.1 | hypothetical protein | none |
|  | A6P53_10395 | AND73232.1 | hypothetical protein | none |
|  | A6P53_10400 | AND73233.1 | hypothetical protein | none |
|  | A6P53_10405 | AND73234.1 | hypothetical protein | T |
|  | A6P53_10410 | AND73235.1 | hypothetical protein | L |
|  | A6P53_10415 | AND73236.1 | hypothetical protein | none |
|  | A6P53_10425 | AND73238.1 | hypothetical protein | none |
|  | A6P53_10440 | AND73241.1 | hypothetical protein | M |
|  | A6P53_10450 | AND73243.1 | hypothetical protein | none |
|  | A6P53_10460 | AND73245.1 | hypothetical protein | none |
| Region #5 | A6P53_10470 | AND73247.1 | group 1 glycosyl transferase | none |
|  | A6P53_10475 | AND73248.1 | hypothetical protein | S |
|  | A6P53_10480 | AND73249.1 | hypothetical protein | none |
|  | A6P53_10485 | AND73250.1 | glycosyl transferase | M |
|  | A6P53_10490 | AND73251.1 | hypothetical protein | M |
|  | A6P53_10625 | AND73276.1 | hypothetical protein | none |
|  | A6P53_11260 | AND73393.1 | hypothetical protein | none |
|  | A6P53_11280 | AND73733.1 | guanosine 5'-monophosphate oxidoreductase | V |
|  | A6P53_11330 | AND73402.1 | glucose-6-phosphate isomerase | GR |
|  | A6P53_11335 | AND73403.1 | mannose-6-phosphate isomerase | G |
|  | A6P53_11340 | AND73404.1 | hypothetical protein | G |
|  | A6P53_11355 | AND73407.1 | PTS system sorbose subfamily IIB component | G |
|  | A6P53_11360 | AND73408.1 | GntR family transcriptional regulator | K |
|  | A6P53_11475 | AND73735.1 | hypothetical protein | none |
|  | A6P53_11675 | AND73737.1 | cell wall hydrolase | none |
|  | A6P53_12510 | AND73601.1 | type III restriction endonuclease subunit R | KL |
|  | A6P53_12515 | AND73602.1 | adenine methyltransferase | V |
|  | A6P53_12525 | AND73603.1 | hypothetical protein | none |
|  | A6P53_12800 | AND73746.1 | beta-ketoacyl-[acyl-carrier-protein] synthase II | IQ |

^*^ Region contains more than five consecutive strain-specific genes.

**Supplementary Table 3.** *E. hirae* R17 genomic islands and genes.

| **R17 protein ID (Chromosome/plasmid)** | **Start** | **Stop** | **Length(bp)** | **Strand** | **Gene Name** | **Gene Product Name** |
| --- | --- | --- | --- | --- | --- | --- |
| GI1(Chromosome) | 64767 | 80239 | 15,473 |  |  |  |
| [AND71368.1](https://www.ncbi.nlm.nih.gov/protein/1026744001) | 64767 | 65666 | 900 | + | - | D-tagatose-1,6-bisphosphate aldolase subunit GatY |
| [AND71369.1](https://www.ncbi.nlm.nih.gov/protein/1026744001) | 66126 | 66434 | 309 | + | - | 30S ribosomal protein S10 |
| [AND71370.1](https://www.ncbi.nlm.nih.gov/protein/1026744001) | 66465 | 67094 | 630 | + | - | 50S ribosomal protein L3 |
| [AND71371.1](https://www.ncbi.nlm.nih.gov/protein/1026744001) | 67122 | 67745 | 624 | + | - | 50S ribosomal protein L4 |
| [AND71372.1](https://www.ncbi.nlm.nih.gov/protein/1026744001) | 67745 | 68035 | 291 | + | - | 50S ribosomal protein L23 |
| [AND71373.1](https://www.ncbi.nlm.nih.gov/protein/1026744001) | 68075 | 68908 | 834 | + | *rplB* | 50S ribosomal protein L2 |
| [AND71374.1](https://www.ncbi.nlm.nih.gov/protein/1026744001) | 68951 | 69229 | 279 | + | - | 30S ribosomal protein S19 |
| [AND71375.1](https://www.ncbi.nlm.nih.gov/protein/1026744001) | 69251 | 69598 | 348 | + | - | 50S ribosomal protein L22 |
| [AND71376.1](https://www.ncbi.nlm.nih.gov/protein/1026744001) | 69612 | 70268 | 657 | + | - | 30S ribosomal protein S3 |
| [AND71377.1](https://www.ncbi.nlm.nih.gov/protein/1026744001) | 70271 | 70705 | 435 | + | - | 50S ribosomal protein L16 |
| [AND71378.1](https://www.ncbi.nlm.nih.gov/protein/1026744001) | 70695 | 70883 | 189 | *+* | - | 50S ribosomal protein L29 |
| [AND71379.1](https://www.ncbi.nlm.nih.gov/protein/1026744001) | 70908 | 71174 | 267 | + | - | 30S ribosomal protein S17 |
| [AND71380.1](https://www.ncbi.nlm.nih.gov/protein/1026744001) | 71232 | 71600 | 369 | + | - | 50S ribosomal protein L14 |
| [AND71381.1](https://www.ncbi.nlm.nih.gov/protein/1026744001) | 71637 | 71948 | 312 | + | - | 50S ribosomal protein L24 |
| [AND71382.1](https://www.ncbi.nlm.nih.gov/protein/1026744001) | 71974 | 72513 | 540 | + | - | 50S ribosomal protein L5 |
| [AND71383.1](https://www.ncbi.nlm.nih.gov/protein/1026744001) | 72532 | 72717 | 186 | + | *rpsN* | 30S ribosomal protein S14 type Z |
| [AND71384.1](https://www.ncbi.nlm.nih.gov/protein/1026744001) | 72754 | 73152 | 399 | + | - | 30S ribosomal protein S8 |
| [AND71385.1](https://www.ncbi.nlm.nih.gov/protein/1026744001) | 73184 | 73720 | 537 | + | - | 50S ribosomal protein L6 |
| [AND73657.1](https://www.ncbi.nlm.nih.gov/protein/1026746290) | 73879 | 74235 | 357 | + | - | 50S ribosomal protein L18 |
| [AND71386.1](https://www.ncbi.nlm.nih.gov/protein/1026744001) | 74256 | 74756 | 501 | + | - | 30S ribosomal protein S5 |
| [AND71387.1](https://www.ncbi.nlm.nih.gov/protein/1026744001) | 74771 | 74950 | 180 | + | - | 50S ribosomal protein L30 |
| [AND71388.1](https://www.ncbi.nlm.nih.gov/protein/1026744001) | 75012 | 75452 | 441 | *+* | - | 50S ribosomal protein L15 |
| [AND71389.1](https://www.ncbi.nlm.nih.gov/protein/1026744001) | 75452 | 76747 | 1,296 | + | - | Protein translocase subunit SecY |
| [AND71390.1](https://www.ncbi.nlm.nih.gov/protein/1026744001) | 76807 | 77454 | 648 | + | - | Adenylate kinase |
| [AND71391.1](https://www.ncbi.nlm.nih.gov/protein/1026744001) | 77646 | 77864 | 219 | + | *infA* | Translation initiation factor IF-1 |
| [AND71392.1](https://www.ncbi.nlm.nih.gov/protein/1026744001) | 77897 | 78013 | 117 | + | - | 50S ribosomal protein L36 |
| [AND71393.1](https://www.ncbi.nlm.nih.gov/protein/1026744001) | 78031 | 78396 | 366 | + | - | 30S ribosomal protein S13 |
| [AND71394.1](https://www.ncbi.nlm.nih.gov/protein/1026744001) | 78424 | 78813 | 390 | + | - | 30S ribosomal protein S11 |
| [AND71395.1](https://www.ncbi.nlm.nih.gov/protein/1026744001) | 78893 | 79831 | 939 | + | - | DNA-directed RNA polymerase subunit alpha |
| [AND71396.1](https://www.ncbi.nlm.nih.gov/protein/1026744029) | 79859 | 80239 | 381 | + | - | 50S ribosomal protein L17 |
| GI2(Chromosome) | 955334 | 972444 | 17,111 |  |  |  |
| [AND72116.1](https://www.ncbi.nlm.nih.gov/protein/1026744749) | 955147 | 961971 | 6,825 | + | - | Hypothetical protein |
| A6P53_04350(locus_tag) | 962117 | 967600 | 5,484 | + | - | Hypothetical protein/Pseudogene |
| [AND72117.1](https://www.ncbi.nlm.nih.gov/protein/1026744749) | 967673 | 969127 | 1,455 | + | - | Hypothetical protein |
| [AND72118.1](https://www.ncbi.nlm.nih.gov/protein/1026744749) | 969276 | 972998 | 3,723 | + | - | Hypothetical protein |
| GI3(Chromosome) | 1588927 | 1598660 | 9,734 |  |  |  |
| [AND72645.1](https://www.ncbi.nlm.nih.gov/protein/1026745278) | 1588927 | 1590420 | 1,494 | + | - | Uncharacterized transcriptional regulatory protein YesN |
| [AND72646.1](https://www.ncbi.nlm.nih.gov/protein/1026745278) | 1590395 | 1592107 | 1,713 | + | - | Sensor histidine kinase YesM |
| [AND72647.1](https://www.ncbi.nlm.nih.gov/protein/1026745278) | 1592407 | 1593360 | 954 | - | - | Beta-galactosidase small subunit |
| [AND72648.1](https://www.ncbi.nlm.nih.gov/protein/1026745278) | 1593341 | 1595218 | 1,878 | - | - | Beta-galactosidase large subunit |
| [AND72649.1](https://www.ncbi.nlm.nih.gov/protein/1026745278) | 1595223 | 1597460 | 2,238 | - | - | Alpha-galactosidase 1 |
| [AND72650.1](https://www.ncbi.nlm.nih.gov/protein/1026745278) | 1597576 | 1598607 | 1,032 | + | - | Uncharacterized HTH-type transcriptional regulator MsmR |
| GI4(Chromosome) | 1787175 | 1790973 | 3,799 |  |  |  |
| [AND72787.1](https://www.ncbi.nlm.nih.gov/protein/1026745420) | 1787478 | 1787984 | 507 | + | - | Hypothetical protein |
| [AND72788.1](https://www.ncbi.nlm.nih.gov/protein/1026745420) | 1788098 | 1788454 | 357 | + | - | Hypothetical protein |
| A6P53_07950 (locus_tag) | 1788563 | 1789276 | 714 | + | - | Hypothetical protein/Pseudogene |
| [AND72789.1](https://www.ncbi.nlm.nih.gov/protein/1026745420) | 1789294 | 1789776 | 483 | + | - | Hypothetical protein |
| [AND72790.1](https://www.ncbi.nlm.nih.gov/protein/1026745420) | 1789880 | 1790080 | 201 | + | - | Hypothetical protein |
| [AND72791.1](https://www.ncbi.nlm.nih.gov/protein/1026745420) | 1790101 | 1790973 | 873 | + | - | Hypothetical protein |
| GI5(Chromosome) | 1829310 | 1833381 | 4,072 |  |  |  |
| [AND72822.1](https://www.ncbi.nlm.nih.gov/protein/1026745456) | 1829310 | 1829441 | 132 | - | - | Hypothetical protein |
| [AND72823.1](https://www.ncbi.nlm.nih.gov/protein/1026745456) | 1829524 | 1829817 | 294 | - | - | Hypothetical protein |
| [AND72824.1](https://www.ncbi.nlm.nih.gov/protein/1026745456) | 1830180 | 1830674 | 495 | + | - | Hypothetical protein |
| [AND72825.1](https://www.ncbi.nlm.nih.gov/protein/1026745456) | 1830975 | 1831442 | 468 | - | - | Uncharacterized phage-related protein Lin1259/Lin1 739 |
| [AND72826.1](https://www.ncbi.nlm.nih.gov/protein/1026745456) | 1831712 | 1832401 | 690 | + | - | Hypothetical protein |
| [AND72827.1](https://www.ncbi.nlm.nih.gov/protein/1026745456) | 1832501 | 1832998 | 498 | - | - | Hypothetical protein |
| [AND72828.1](https://www.ncbi.nlm.nih.gov/protein/1026745456) | 1833166 | 1833381 | 216 | + | - | Hypothetical protein |
| GI6(Chromosome) | 1837643 | 1843264 | 5,622 |  |  |  |
| [AND72836.1](https://www.ncbi.nlm.nih.gov/protein/1026745469) | 1837684 | 1837869 | 186 | - | - | Hypothetical protein |
| [AND72837.1](https://www.ncbi.nlm.nih.gov/protein/1026745469) | 1837886 | 1838116 | 231 | - | - | Hypothetical protein |
| [AND72838.1](https://www.ncbi.nlm.nih.gov/protein/1026745469) | 1838113 | 1838667 | 555 | - | - | Hypothetical protein |
| [AND72839.1](https://www.ncbi.nlm.nih.gov/protein/1026745469) | 1838955 | 1839782 | 828 | + | - | Hypothetical protein |
| [AND72840.1](https://www.ncbi.nlm.nih.gov/protein/1026745469) | 1839723 | 1839974 | 252 | - | - | Hypothetical protein |
| [AND72841.1](https://www.ncbi.nlm.nih.gov/protein/1026745469) | 1839986 | 1840225 | 240 | - | - | Hypothetical protein |
| [AND72842.1](https://www.ncbi.nlm.nih.gov/protein/1026745469) | 1840364 | 1840756 | 393 | + | - | HTH-type transcriptional repressor RghR |
| [AND72843.1](https://www.ncbi.nlm.nih.gov/protein/1026745469) | 1840763 | 1841191 | 429 | + | - | Uncharacterized immunity region protein 2/toxin |
| [AND72844.1](https://www.ncbi.nlm.nih.gov/protein/1026745469) | 1841253 | 1841882 | 630 | + | - | Hypothetical protein |
| [AND72845.1](https://www.ncbi.nlm.nih.gov/protein/1026745469) | 1842041 | 1843264 | 1,224 | + | - | Tyrosine recombinase XerC-like |
| GI7(Chromosome) | 1866415 | 1880351 | 13,937 |  |  |  |
| [AND72865.1](https://www.ncbi.nlm.nih.gov/protein/1026745498) | 1866415 | 1866642 | 228 | + | - | Hypothetical protein |
| A6P53_08380 | 1866711 | 1867133 | 423 | + | - | Transposase |
| [AND72866.1](https://www.ncbi.nlm.nih.gov/protein/1026745499) | 1867319 | 1867654 | 336 | - | - | Hypothetical protein |
| [AND72867.1](https://www.ncbi.nlm.nih.gov/protein/1026745500) | 1867644 | 1868519 | 876 | - | - | Hypothetical protein |
| A6P53_08395 | 1868516 | 1869364 | 849 | - | - | Hypothetical protein/Pseudogene |
| [AND72868.1](https://www.ncbi.nlm.nih.gov/protein/1026745501) | 1869361 | 1869582 | 222 | - | - | Hypothetical protein |
| A6P53_08405 | 1870047 | 1874462 | 4,416 | - | - | Hypothetical protein/Pseudogene |
| A6P53_08410 | 1875025 | 1875456 | 432 | - | - | Hypothetical protein/Pseudogene |
| [AND72869.1](https://www.ncbi.nlm.nih.gov/protein/1026745502) | 1875935 | 1877941 | 2,007 | - | - | Hypothetical protein |
| [AND72870.1](https://www.ncbi.nlm.nih.gov/protein/1026745503) | 1877928 | 1878542 | 615 | - | - | Putative transposon Tn*552* DNA-invertase bin3 |
| A6P53_08425 | 1879231 | 1879761 | 531 | - | - | Hypothetical protein/Pseudogene |
| [AND72871.1](https://www.ncbi.nlm.nih.gov/protein/1026745504) | 1880028 | 1880351 | 324 | + | - | Hypothetical protein |
| A6P53_08435 | 1880314 | 1880745 | 432 | + | - | Transposase |
| GI8(Chromosome) | 1895845 | 1903312 | 7,468 |  |  |  |
| [AND73716.1](https://www.ncbi.nlm.nih.gov/protein/1026746349) | 1895599 | 1898181 | 2,583 | - | - | Sensor protein KdpD |
| [AND73717.1](https://www.ncbi.nlm.nih.gov/protein/1026746349) | 1898267 | 1898800 | 534 | - | - | Potassium-transporting ATPase C chain |
| [AND73718.1](https://www.ncbi.nlm.nih.gov/protein/1026746349) | 1898813 | 1900843 | 2,031 | - | - | Potassium-transporting ATPase B chain |
| [AND73719.1](https://www.ncbi.nlm.nih.gov/protein/1026746349) | 1900853 | 1902529 | 1,677 | - | - | Potassium-transporting ATPase A chain |
| [AND73720.1](https://www.ncbi.nlm.nih.gov/protein/1026746349) | 1902839 | 1903804 | 966 | - | - | Tagatose 1,6-diphosphate aldolase 1 |
| GI9(Chromosome) | 2304635 | 2319535 | 14,901 |  |  |  |
| [AND73226.1](https://www.ncbi.nlm.nih.gov/protein/1026745859) | 2304674 | 2305111 | 438 | - | - | Hypothetical protein |
| [AND73227.1](https://www.ncbi.nlm.nih.gov/protein/1026745859) | 2305348 | 2306130 | 783 | - | - | Hypothetical protein |
| [AND73228.1](https://www.ncbi.nlm.nih.gov/protein/1026745859) | 2306147 | 2306803 | 657 | - | - | Uncharacterized protein YhcS |
| [AND73229.1](https://www.ncbi.nlm.nih.gov/protein/1026745859) | 2306807 | 2307475 | 669 | - | - | Hypothetical protein |
| [AND73230.1](https://www.ncbi.nlm.nih.gov/protein/1026745859) | 2307710 | 2307976 | 267 | + | - | Hypothetical protein |
| [AND73231.1](https://www.ncbi.nlm.nih.gov/protein/1026745859) | 2308199 | 2313919 | 5,721 | - | - | Viral-enhancing factor |
| [AND73232.1](https://www.ncbi.nlm.nih.gov/protein/1026745859) | 2314337 | 2315800 | 1,464 | + | - | Hypothetical protein |
| [AND73233.1](https://www.ncbi.nlm.nih.gov/protein/1026745859) | 2316076 | 2317491 | 1,416 | - | - | Hypothetical protein |
| [AND73234.1](https://www.ncbi.nlm.nih.gov/protein/1026745859) | 2317935 | 2318525 | 591 | - | - | Arabinose operon regulatory protein |
| [AND73235.1](https://www.ncbi.nlm.nih.gov/protein/1026745859) | 2318536 | 2318733 | 198 | - | - | Hypothetical protein |
| [AND73236.1](https://www.ncbi.nlm.nih.gov/protein/1026745859) | 2319074 | 2319265 | 192 | + | - | Hypothetical protein |
| [AND73237.1](https://www.ncbi.nlm.nih.gov/protein/1026745859) | 2319680 | 2320975 | 1,296 | + | - | Transposase |
| GI10(Chromosome) | 2321054 | 2332996 | 11,943 |  |  |  |
| [AND73238.1](https://www.ncbi.nlm.nih.gov/protein/1026745871) | 2321098 | 2321304 | 207 | - | - | Hypothetical protein |
| [AND73239.1](https://www.ncbi.nlm.nih.gov/protein/1026745871) | 2321353 | 2325411 | 4059 | - | - | Hypothetical protein |
| [AND73240.1](https://www.ncbi.nlm.nih.gov/protein/1026745871) | 2325810 | 2327120 | 1311 | - | - | UDP-N-acetyl-D-glucosamine 6-dehydrogenase |
| [AND73241.1](https://www.ncbi.nlm.nih.gov/protein/1026745871) | 2327137 | 2328411 | 1275 | - | - | Hyaluronan synthase |
| [AND73242.1](https://www.ncbi.nlm.nih.gov/protein/1026745871) | 2328824 | 2329000 | 177 | - | - | Hypothetical protein |
| [AND73243.1](https://www.ncbi.nlm.nih.gov/protein/1026745871) | 2329077 | 2331086 | 2010 | - | - | N-acetylmuramoyl-L-alanine amidase domain-containing protein SAOUHSC_02979 |
| GI11(Chromosome) | 2333043 | 2338664 | 5,622 |  |  |  |
| [AND73243.1](https://www.ncbi.nlm.nih.gov/protein/1026745876) | 2332043 | 2333182 | 1,140 | - | - | Hypothetical protein |
| [AND73244.1](https://www.ncbi.nlm.nih.gov/protein/1026745876) | 2333157 | 2334278 | 1,122 | - | - | UDP-N-acetylglucosamine 2-epimerase |
| [AND73245.1](https://www.ncbi.nlm.nih.gov/protein/1026745876) | 2334295 | 2334879 | 585 | - | - | Teichoic acids export ATP-binding protein TagH |
| [AND73246.1](https://www.ncbi.nlm.nih.gov/protein/1026745876) | 2335174 | 2335983 | 810 | - | - | Teichoic acid translocation permease protein TagG |
| [AND73247.1](https://www.ncbi.nlm.nih.gov/protein/1026745876) | 2336013 | 2338157 | 2,145 | - | - | Hypothetical protein |
| [AND73248.1](https://www.ncbi.nlm.nih.gov/protein/1026745876) | 2338176 | 2340320 | 2,145 | - | - | Spore protein YkvP |
| GI12(Chromosome) | 2338681 | 2343421 | 4,741 |  |  |  |
| [AND73248.1](https://www.ncbi.nlm.nih.gov/protein/1026745881) | 2338176 | 2340320 | 2145 | - | - | Spore protein YkvP |
| [AND73249.1](https://www.ncbi.nlm.nih.gov/protein/1026745881) | 2340328 | 2340693 | 366 | - | - | Hypothetical protein |
| [AND73250.1](https://www.ncbi.nlm.nih.gov/protein/1026745881) | 2340705 | 2341934 | 1230 | - | - | Uncharacterized glycosyltransferase MJ1607 |
| [AND73251.1](https://www.ncbi.nlm.nih.gov/protein/1026745881) | 2341936 | 2343132 | 1197 | - | - | Hypothetical protein |
| [AND73252.1](https://www.ncbi.nlm.nih.gov/protein/1026745881) | 2343126 | 2344412 | 1287 | - | - | UDP-N-acetyl-D-mannosamine dehydrogenase |
| GIP1(Plasmid) | 28335 | 36106 | 7,772 |  |  |  |
| [AND73773.1](https://www.ncbi.nlm.nih.gov/protein/1026746407) | 28335 | 28616 | 282 | + | - | Plasmid recombination enzyme type 2 |
| [AND73774.1](https://www.ncbi.nlm.nih.gov/protein/1026746407) | 28639 | 29319 | 681 | - | - | Transposase for insertion sequence-like element IS*1216E* |
| [AND73775.1](https://www.ncbi.nlm.nih.gov/protein/1026746407) | 30253 | 30990 | 738 | - | - | rRNA adenine N-6-methyltransferase |
| [AND73776.1](https://www.ncbi.nlm.nih.gov/protein/1026746407) | 31115 | 31198 | 84 | - | - | 23S rRNA methyltransferase |
| [AND73777.1](https://www.ncbi.nlm.nih.gov/protein/1026746407) | 31420 | 32106 | 687 | + | - | Transposase for insertion sequence-like element IS*Enfa1* |
| [AND73778.1](https://www.ncbi.nlm.nih.gov/protein/1026746407) | 32303 | 33133 | 831 | - | - | Undecaprenyl-diphosphatase (BcrD) |
| [AND73779.1](https://www.ncbi.nlm.nih.gov/protein/1026746407) | 33133 | 33882 | 750 | - | - | Bacitracin transport permease protein BcrB |
| [AND73780.1](https://www.ncbi.nlm.nih.gov/protein/1026746407) | 33875 | 34792 | 918 | - | - | Bacitracin transport ATP-binding protein BcrA |
| [AND73781.1](https://www.ncbi.nlm.nih.gov/protein/1026746407) | 34975 | 35589 | 615 | - | - | Cryptic phage CTXphi transcriptional repressor BcrR |
| [AND73782.1](https://www.ncbi.nlm.nih.gov/protein/1026746407) | 35579 | 35776 | 198 | - | - | Hypothetical protein |

**Supplementary Table S4.** Virulence factors identified in *E. hirae* R17

| **Protein ID** | **Predicted Protein** | **Subject or Function (well-characterized origin strain)** | **Identities (%)** | **Match (%)** |
| --- | --- | --- | --- | --- |
| [AND71552.1](https://www.ncbi.nlm.nih.gov/protein/1026744185) | BopD | LacI family sugar-binding transcriptional regulator; Homologous to a sugar-binding transcriptional regulator involved in biofilm production. (*Enterococcus faecium* Aus0085) | 88.32 | 98.24 |
| [AND72745.1](https://www.ncbi.nlm.nih.gov/protein/1026745378) | UppS | Undecaprenyl diphosphate synthase (*Enterococcus faecium* Aus0004) | 88.15 | 100 |
| [AND73037.1](https://www.ncbi.nlm.nih.gov/protein/1026745670) | Eno | Phosphopyruvate hydratase (*Streptococcus pneumoniae* D39) | 83.14 | 100 |
| [AND73144.1](https://www.ncbi.nlm.nih.gov/protein/1026745777) | ClpP | ATP-dependent Clp protease proteolytic subunit; Serine protease involved in proteolysis and is required for growth under stress conditions. (*Listeria monocytogenes* EGD-e) | 81.12 | 99.49 |
| [AND72744.1](https://www.ncbi.nlm.nih.gov/protein/1026745377) | EFAU085_01747 | Phosphatidate cytidylyltransferase; capsule (*Enterococcus faecium* Aus0085) | 80.45 | 100 |
| [AND71992.1](https://www.ncbi.nlm.nih.gov/protein/1026744625) | RmlB | Putative dTDP-glucose-4,6-dehydratase (*Streptococcus mutans* UA159) | 80.31 | 95.03 |
| [AND71990.1](https://www.ncbi.nlm.nih.gov/protein/1026744623) | RmlA | Putative glucose-1-phosphate thymidylytransferase; capsule (*Streptococcus sanguinis* SK36) | 79.65 | 98.96 |
| [AND73040.1](https://www.ncbi.nlm.nih.gov/protein/1026745673) | Plr/GapA | Glyceraldehyde-3-phosphate dehydrogenase, type I (*Streptococcus pneumoniae* Hungary19A-6) | 76.88 | 100 |
| [AND72989.1](https://www.ncbi.nlm.nih.gov/protein/1026745622) | SMU.322c | Glucose-1-phosphate uridylyltransferase (*Streptococcus mutans* UA159) | 76.43 | 95.19 |
| [AND72094.1](https://www.ncbi.nlm.nih.gov/protein/1026744727) | LisR/LisK | Two-component response regulator (*Listeria monocytogenes* EGD-e) | 72.81 | 99.12 |
| [AND73197.1](https://www.ncbi.nlm.nih.gov/protein/1026745830) | SMU.322c | Glucose-1-phosphate uridylyltransferase (*Streptococcus mutans* UA159) | 72.6 | 100 |
| [AND73314.1](https://www.ncbi.nlm.nih.gov/protein/1026745947) | EfaA | Periplasmic solute binding protein; Might be functioning as an adhesin in endocarditis. A solute binding-protein receptor for manganese transport system. (*Enterococcus faecium* Aus0004) | 71.2 | 99.37 |
| [AND72142.1](https://www.ncbi.nlm.nih.gov/protein/1026744775) | EF-Tu | Surface-expressed elongation factor-Tu (EF-Tu) mediates attachment by interacting with host cell nucleolin (*Mycoplas mamycoides* subsp. *mycoides* SC str. PG1) | 70.3 | 99.75 |
| [AND71707.1](http://www.ncbi.nlm.nih.gov/protein/1026744340) | GroEL | Chaperonin; Dispensable for bacterial survival and growth in macrophage infection (*Clostridium thermocel* lum ATCC 27405) | 69.77 | 95.38 |
| [AND72530.1](http://www.ncbi.nlm.nih.gov/protein/1026745163) | Bsh | Bile salt hydrolase; Involved in resisting the acute toxicity of bile and bile salts, important for intestinal persistence (*Listeria seeligeri* serovar 1/2b str. SLCC3954) | 69.54 | 100 |
| [AND71360.1](http://www.ncbi.nlm.nih.gov/protein/1026743993) | EF-Tu | Surface-expressed elongation factor-Tu (EF-Tu) mediates attachment by interacting with host cell nucleolin (*Mycoplasma mycoides* subsp. *mycoides* SC str. PG1) | 69.04 | 99.75 |
| [AND71602.1](http://www.ncbi.nlm.nih.gov/protein/1026744235) | PilB | PilB-type pili (*Enterococcus faecium* str. E1165) | 67.68 | 99.68 |
| [AND72952.1](http://www.ncbi.nlm.nih.gov/protein/1026745585) | BCE_5394 | Aminotransferase family protein, polysaccharide capsule (*Bacillus cereus* ATCC 10987) | 67.49 | 97.58 |
| [AND71526.1](http://www.ncbi.nlm.nih.gov/protein/1026744159) | Lap | Aldehyde-alcohol dehydrogenase protein; Promotes bacterial adhesion to intestinal cells (*Listeria welshimeri* serovar 6b str. SLCC5334) | 66.09 | 99.54 |
| [AND72768.1](http://www.ncbi.nlm.nih.gov/protein/1026745401) | GalE | UDP-glucose 4-epimerase, polysaccharide capsule (*Bacillus thuringiensis* serovar konkukian str. 97-27) | 63.41 | 99.70 |
| [AND72676.1](http://www.ncbi.nlm.nih.gov/protein/1026745309) | Tig/RopA | Trigger factor (*Streptococcus pneumoniae* TIGR4) | 62.91 | 99.30 |
| [AND71456.1](http://www.ncbi.nlm.nih.gov/protein/1026744089) | MsrA/B (PilB) | Trifunctional thioredoxin/methionine sulfoxide reductase A/B protein (*Neisseria meningitidis* Z2491) | 62.7 | 85.71 |
| [AND72698.1](http://www.ncbi.nlm.nih.gov/protein/1026745331) | MOMP | Molecular chaperone DnaK; A putative porin and a multifuction surface protein, may play an important role in adherence (*Chlamydia trachomatis* D/UW-3/CX) | 61.95 | 89.02 |
| [AND71918.1](http://www.ncbi.nlm.nih.gov/protein/1026744551) | LplA1 | Putative lipoate protein ligase A; Necessary for efficient intracellular proliferation (*Listeria ivanovii* subsp. *ivanovii* PAM 55) | 61.79 | 99.10 |
| [AND71993.1](http://www.ncbi.nlm.nih.gov/protein/1026744626) | SMU.824 | dTDP-4-keto-L-rhamnose reductase (*Streptococcus mutans* UA159) | 61.68 | 89.54 |
| [AND72045.1](http://www.ncbi.nlm.nih.gov/protein/1026744678) | ClpE | ATP-dependent protease; An ATPase required for prolonged survival at 42 degree. Acts synergistically with ClpC in cell division (*Listeria monocytogenes* EGD-e) | 60.63 | 96.51 |
| [AND72949.1](http://www.ncbi.nlm.nih.gov/protein/1026745582) | WbfV/WcvB | Predicted UDP-glucose 6-dehydrogenase (*Vibrio vulnificus* CMCP6) | 59.28 | 99.74 |
| [AND71603.1](http://www.ncbi.nlm.nih.gov/protein/1026744236) | Ebp pili | Sortase; Ebp pili (*Enterococcus faecalis* OG1RF) | 58.87 | 90.18 |
| [AND71519.1](http://www.ncbi.nlm.nih.gov/protein/1026744152) | EpsE | Sugar transferase; probable phospho-glucosyltransferase; polysaccharide capsule (*Bacillus thuringiensis* serovar *konkukian* str. 97-27) | 58.72 | 77.13 |
| [AND73636.1](http://www.ncbi.nlm.nih.gov/protein/1026746269) | ClpC | Endopeptidase Clp ATP-binding chain C; An ATPase promoting early escape form the phagosome of macrophages. ClpC is also required for adhesion and invasion, possibly by modulating the expression of InlA, InlB and ActA (*Listeria monocytogenes* EGD-e) | 58.54 | 96.25 |
| [AND72043.1](http://www.ncbi.nlm.nih.gov/protein/1026744676) | SPH_0465 | UDP-N-acetylglucosamine 2-epimerase; capsule (*Streptococcus pneumoniae* Hungary19A-6) | 58.17 | 98.09 |
| [AND72951.1](http://www.ncbi.nlm.nih.gov/protein/1026745584) | PglC | General glycosylation pathway protein; N-linked protein glycosylation (*Campylobacter fetus* subsp. *fetus* 82-40) | 57.99 | 81.25 |
| [AND71600.1](http://www.ncbi.nlm.nih.gov/protein/1026744233) | Ebp pili | Endocarditis and biofilm-associated pilus subunitA; Ebp pili (*Enterococcus faecalis* D32) | 57.5 | 88.01 |
| [AND73252.1](http://www.ncbi.nlm.nih.gov/protein/1026745885) | Cap5O | Capsular polysaccharide biosynthesis protein Cap5O; Capsule (*Staphylococcus aureus* subsp. *aureus* USA300_FPR3757) | 56.76 | 96.73 |
| [AND72376.1](http://www.ncbi.nlm.nih.gov/protein/1026745009) | Plr/GapA | Glyceraldehyde-3-phosphate dehydrogenase; Streptococca plasmin receptor/GAPDH (*Streptococcus agalactiae* A909) | 56.51 | 99.40 |
| [AND72041.1](http://www.ncbi.nlm.nih.gov/protein/1026744674) | EF3023 | Polysaccharide lyase, family 8; Hyaluronidase (*Enterococcus faecalis* V583) | 56.15 | 98.98 |
| [AND72373.1](http://www.ncbi.nlm.nih.gov/protein/1026745006) | SlrA | Peptidyl-prolyl cis-trans isomerase, cyclophilin-type; Streptococcal lipoprotein rotamase A (*Streptococcus pneumoniae* CGSP14) | 55.22 | 79.76 |
| [AND72059.1](http://www.ncbi.nlm.nih.gov/protein/1026744692) | FabZ | (3R)-hydroxymyristoyl ACP dehydratase (*Brucella suis* 1330) | 54.61 | 100 |
| [AND72149.1](http://www.ncbi.nlm.nih.gov/protein/1026744782) | SigA/RpoV | RNA polymerase sigma factor A; Sigma A interacts with a transcriptional activator WhiB3 to allow the expression of genes necessary for virulence (*Mycobacterium tuberculosis* RGTB423) | 54.41 | 87.80 |
| [AND73298.1](http://www.ncbi.nlm.nih.gov/protein/1026745931) | VirR/VirS | Hypothetical protein (*Listeria monocytogenes* EGD-e) | 54.02 | 100 |
| [AND73400.1](http://www.ncbi.nlm.nih.gov/protein/1026746033) | HtrA/DegP | Serine peptidase (*Streptococcus agalactiae* A909) | 53.64 | 86.21 |
| [AND72388.1](http://www.ncbi.nlm.nih.gov/protein/1026745021) | Aut | N-acetylmuramoyl-L-alanine amidase family protein; Required for entry of *L. monocytogenes* into nonphagocytic cells and necessary for full virulence (*Listeria seeligeri* serovar 1/2b str. SLCC3954) | 52.2 | 55.05 |
| [AND71943.1](http://www.ncbi.nlm.nih.gov/protein/1026744576) | CD1208 | Putative RNA methyltransferase; Hemolysin (*Clostridium difficile* 630) | 51.49 | 97.79 |
| [AND73526.1](http://www.ncbi.nlm.nih.gov/protein/1026746159) | Stp | Putative phosphoprotein phosphatase; Serine-threonine phosphatase (*Listeria ivanovii* subsp. *ivanovii* PAM 55) | 51.04 | 97.97 |
| [AND71427.1](http://www.ncbi.nlm.nih.gov/protein/1026744060) | VctC | Iron(III) ABC transporter, ATP-binding protein, periplasmi c binding protein-dependent ABC transport systems (*Vibrio cholerae* O1 biovar El Tor str. N16961) | 50.8 | 99.60 |
| [AND72156.1](http://www.ncbi.nlm.nih.gov/protein/1026744789) | ScpB | Segregation and condensation protein B; Fibronectin-bindin g protein (*Streptococcus agalactiae* 2603V/R) | 50.33 | 78.53 |
| [AND73216.1](http://www.ncbi.nlm.nih.gov/protein/1026745849) | SugC | Carbohydrate ABC transporter ATP-binding protein, CUT1 family; Trehalose-recycling ABC transporter (*Mycobacterium smegmatis* JS623) | 50.26 | 100 |

**Supplementary Table 5. Tandem repeats identified in *E．hirae* R17**

| Indices | Period Size | Copy Number | Consensus Size | Percent Matches | Percent Indels | Score | A | C | G | T | Entropy (0-2) |
| --- | --- | --- | --- | --- | --- | --- | --- | --- | --- | --- | --- |
| **Chromosome** |  |  |  |  |  |  |  |  |  |  |  |
| [34419--34455](http://tandem.bu.edu/trf/output/45aYwQQncxAmY.2.7.7.80.10.50.500.1.txt.html#34419--34455,7,5.3,7,1) | 7 | 5.3 | 7 | 100 | 0 | 74 | 72 | 13 | 13 | 0 | 1.11 |
| [84219--84862](http://tandem.bu.edu/trf/output/45aYwQQncxAmY.2.7.7.80.10.50.500.1.txt.html#84219--84862,201,3.2,201,4) | 201 | 3.2 | 201 | 93 | 1 | 1121 | 42 | 20 | 16 | 21 | 1.89 |
| [85089--85284](http://tandem.bu.edu/trf/output/45aYwQQncxAmY.2.7.7.80.10.50.500.1.txt.html#85089--85284,72,2.7,72,5) | 72 | 2.7 | 72 | 76 | 3 | 216 | 44 | 25 | 15 | 14 | 1.85 |
| [86884--87162](http://tandem.bu.edu/trf/output/45aYwQQncxAmY.2.7.7.80.10.50.500.1.txt.html#86884--87162,99,2.8,99,6) | 99 | 2.8 | 99 | 94 | 0 | 468 | 30 | 13 | 30 | 26 | 1.94 |
| [187122--187162](http://tandem.bu.edu/trf/output/45aYwQQncxAmY.2.7.7.80.10.50.500.1.txt.html#187122--187162,13,3.1,13,7) | 13 | 3.1 | 13 | 86 | 6 | 64 | 58 | 9 | 12 | 19 | 1.61 |
| [218564--219069](http://tandem.bu.edu/trf/output/45aYwQQncxAmY.2.7.7.80.10.50.500.1.txt.html#218564--219069,261,1.9,260,9) | 261 | 1.9 | 260 | 82 | 4 | 617 | 39 | 18 | 20 | 21 | 1.93 |
| [219885--219919](http://tandem.bu.edu/trf/output/45aYwQQncxAmY.2.7.7.80.10.50.500.1.txt.html#219885--219919,18,1.9,18,10) | 18 | 1.9 | 18 | 88 | 0 | 52 | 54 | 14 | 8 | 22 | 1.67 |
| [254434--254801](http://tandem.bu.edu/trf/output/45aYwQQncxAmY.2.7.7.80.10.50.500.1.txt.html#254434--254801,122,3.0,120,12) | 122 | 3.0 | 120 | 76 | 10 | 370 | 30 | 21 | 15 | 32 | 1.94 |
| [263509--263536](http://tandem.bu.edu/trf/output/45aYwQQncxAmY.2.7.7.80.10.50.500.1.txt.html#263509--263536,12,2.3,12,13) | 12 | 2.3 | 12 | 100 | 0 | 56 | 67 | 10 | 14 | 7 | 1.40 |
| [263703--263800](http://tandem.bu.edu/trf/output/45aYwQQncxAmY.2.7.7.80.10.50.500.1.txt.html#263703--263800,12,7.7,12,14) | 12 | 7.7 | 12 | 73 | 13 | 79 | 74 | 3 | 15 | 7 | 1.16 |
| [263705--263788](http://tandem.bu.edu/trf/output/45aYwQQncxAmY.2.7.7.80.10.50.500.1.txt.html#263705--263788,21,3.9,21,15) | 21 | 3.9 | 21 | 74 | 18 | 82 | 72 | 3 | 16 | 7 | 1.21 |
| [263999--264085](http://tandem.bu.edu/trf/output/45aYwQQncxAmY.2.7.7.80.10.50.500.1.txt.html#263999--264085,24,3.6,24,16) | 24 | 3.6 | 24 | 80 | 4 | 86 | 32 | 18 | 25 | 24 | 1.97 |
| [269870--269917](http://tandem.bu.edu/trf/output/45aYwQQncxAmY.2.7.7.80.10.50.500.1.txt.html#269870--269917,21,2.3,21,17) | 21 | 2.3 | 21 | 92 | 0 | 87 | 52 | 27 | 20 | 0 | 1.47 |
| [319478--319511](http://tandem.bu.edu/trf/output/45aYwQQncxAmY.2.7.7.80.10.50.500.1.txt.html#319478--319511,12,2.8,12,18) | 12 | 2.8 | 12 | 95 | 0 | 59 | 44 | 17 | 29 | 8 | 1.79 |
| [320958--321081](http://tandem.bu.edu/trf/output/45aYwQQncxAmY.2.7.7.80.10.50.500.1.txt.html#320958--321081,39,3.2,39,19) | 39 | 3.2 | 39 | 66 | 21 | 105 | 33 | 11 | 24 | 31 | 1.90 |
| [320947--321373](http://tandem.bu.edu/trf/output/45aYwQQncxAmY.2.7.7.80.10.50.500.1.txt.html#320947--321373,78,5.5,78,20) | 78 | 5.5 | 78 | 87 | 2 | 464 | 35 | 13 | 22 | 29 | 1.92 |
| [320992--321120](http://tandem.bu.edu/trf/output/45aYwQQncxAmY.2.7.7.80.10.50.500.1.txt.html#320992--321120,39,3.3,39,21) | 39 | 3.3 | 39 | 64 | 16 | 104 | 34 | 10 | 24 | 31 | 1.90 |
| [321193--321315](http://tandem.bu.edu/trf/output/45aYwQQncxAmY.2.7.7.80.10.50.500.1.txt.html#321193--321315,39,3.2,39,23) | 39 | 3.2 | 39 | 61 | 21 | 94 | 34 | 13 | 23 | 29 | 1.92 |
| [321214--321339](http://tandem.bu.edu/trf/output/45aYwQQncxAmY.2.7.7.80.10.50.500.1.txt.html#321214--321339,39,3.1,42,24) | 39 | 3.1 | 42 | 61 | 21 | 97 | 33 | 14 | 22 | 30 | 1.93 |
| [321814--322143](http://tandem.bu.edu/trf/output/45aYwQQncxAmY.2.7.7.80.10.50.500.1.txt.html#321814--322143,156,2.1,156,26) | 156 | 2.1 | 156 | 87 | 3 | 459 | 36 | 12 | 22 | 29 | 1.90 |
| [322021--322144](http://tandem.bu.edu/trf/output/45aYwQQncxAmY.2.7.7.80.10.50.500.1.txt.html#322021--322144,39,3.2,39,27) | 39 | 3.2 | 39 | 63 | 13 | 101 | 37 | 10 | 20 | 31 | 1.86 |
| [321814--322161](http://tandem.bu.edu/trf/output/45aYwQQncxAmY.2.7.7.80.10.50.500.1.txt.html#321814--322161,78,4.5,78,28) | 78 | 4.5 | 78 | 83 | 4 | 389 | 35 | 11 | 22 | 30 | 1.90 |
| [469488--469526](http://tandem.bu.edu/trf/output/45aYwQQncxAmY.2.7.7.80.10.50.500.1.txt.html#469488--469526,20,2.0,20,30) | 20 | 2.0 | 20 | 89 | 0 | 60 | 51 | 10 | 12 | 25 | 1.71 |
| [473557--474150](http://tandem.bu.edu/trf/output/45aYwQQncxAmY.2.7.7.80.10.50.500.1.txt.html#473557--474150,207,2.9,206,31) | 207 | 2.9 | 206 | 95 | 1 | 1053 | 38 | 19 | 17 | 25 | 1.93 |
| [524897--524957](http://tandem.bu.edu/trf/output/45aYwQQncxAmY.2.7.7.80.10.50.500.1.txt.html#524897--524957,21,2.7,21,32) | 21 | 2.7 | 21 | 82 | 14 | 61 | 11 | 6 | 9 | 72 | 1.29 |
| [527656--527680](http://tandem.bu.edu/trf/output/45aYwQQncxAmY.2.7.7.80.10.50.500.1.txt.html#527656--527680,12,2.1,12,33) | 12 | 2.1 | 12 | 100 | 0 | 50 | 64 | 20 | 8 | 8 | 1.46 |
| [557389--557436](http://tandem.bu.edu/trf/output/45aYwQQncxAmY.2.7.7.80.10.50.500.1.txt.html#557389--557436,12,4.0,12,34) | 12 | 4.0 | 12 | 100 | 0 | 96 | 66 | 16 | 16 | 0 | 1.25 |
| [585087--585292](http://tandem.bu.edu/trf/output/45aYwQQncxAmY.2.7.7.80.10.50.500.1.txt.html#585087--585292,15,13.7,15,36) | 15 | 13.7 | 15 | 94 | 0 | 286 | 44 | 33 | 22 | 0 | 1.57 |
| [596894--597154](http://tandem.bu.edu/trf/output/45aYwQQncxAmY.2.7.7.80.10.50.500.1.txt.html#596894--597154,105,2.5,105,39) | 105 | 2.5 | 105 | 91 | 5 | 420 | 31 | 21 | 19 | 28 | 1.97 |
| [626313--626573](http://tandem.bu.edu/trf/output/45aYwQQncxAmY.2.7.7.80.10.50.500.1.txt.html#626313--626573,121,2.2,121,40) | 121 | 2.2 | 121 | 100 | 0 | 522 | 35 | 13 | 24 | 27 | 1.92 |
| [664259--664839](http://tandem.bu.edu/trf/output/45aYwQQncxAmY.2.7.7.80.10.50.500.1.txt.html#664259--664839,27,21.5,27,41) | 27 | 21.5 | 27 | 83 | 2 | 646 | 38 | 21 | 26 | 13 | 1.91 |
| [664827--664882](http://tandem.bu.edu/trf/output/45aYwQQncxAmY.2.7.7.80.10.50.500.1.txt.html#664827--664882,27,2.1,27,48) | 27 | 2.1 | 27 | 86 | 6 | 78 | 37 | 23 | 23 | 16 | 1.93 |
| [691098--691880](http://tandem.bu.edu/trf/output/45aYwQQncxAmY.2.7.7.80.10.50.500.1.txt.html#691098--691880,411,1.9,411,57) | 411 | 1.9 | 411 | 94 | 2 | 1385 | 34 | 19 | 18 | 27 | 1.95 |
| [755365--755405](http://tandem.bu.edu/trf/output/45aYwQQncxAmY.2.7.7.80.10.50.500.1.txt.html#755365--755405,12,3.4,12,58) | 12 | 3.4 | 12 | 86 | 6 | 57 | 75 | 4 | 19 | 0 | 0.98 |
| [831900--831938](http://tandem.bu.edu/trf/output/45aYwQQncxAmY.2.7.7.80.10.50.500.1.txt.html#831900--831938,20,2.0,20,59) | 20 | 2.0 | 20 | 84 | 0 | 51 | 51 | 0 | 5 | 43 | 1.24 |
| [833481--834522](http://tandem.bu.edu/trf/output/45aYwQQncxAmY.2.7.7.80.10.50.500.1.txt.html#833481--834522,18,58.2,18,60) | 18 | 58.2 | 18 | 81 | 3 | 812 | 16 | 28 | 35 | 19 | 1.93 |
| [833485--834520](http://tandem.bu.edu/trf/output/45aYwQQncxAmY.2.7.7.80.10.50.500.1.txt.html#833485--834520,24,42.9,24,61) | 24 | 42.9 | 24 | 81 | 3 | 779 | 16 | 28 | 35 | 19 | 1.93 |
| [839817--839843](http://tandem.bu.edu/trf/output/45aYwQQncxAmY.2.7.7.80.10.50.500.1.txt.html#839817--839843,12,2.3,12,65) | 12 | 2.3 | 12 | 100 | 0 | 54 | 59 | 33 | 0 | 7 | 1.25 |
| [855343--856014](http://tandem.bu.edu/trf/output/45aYwQQncxAmY.2.7.7.80.10.50.500.1.txt.html#855343--856014,207,3.2,207,71) | 207 | 3.2 | 207 | 96 | 0 | 1220 | 45 | 17 | 21 | 14 | 1.84 |
| [880913--881338](http://tandem.bu.edu/trf/output/45aYwQQncxAmY.2.7.7.80.10.50.500.1.txt.html#880913--881338,156,2.7,153,76) | 156 | 2.7 | 153 | 81 | 5 | 435 | 32 | 13 | 22 | 31 | 1.93 |
| [880913--881295](http://tandem.bu.edu/trf/output/45aYwQQncxAmY.2.7.7.80.10.50.500.1.txt.html#880913--881295,78,4.9,78,78) | 78 | 4.9 | 78 | 81 | 4 | 387 | 33 | 13 | 21 | 31 | 1.92 |
| [881774--882000](http://tandem.bu.edu/trf/output/45aYwQQncxAmY.2.7.7.80.10.50.500.1.txt.html#881774--882000,78,2.9,78,79) | 78 | 2.9 | 78 | 79 | 1 | 204 | 33 | 11 | 22 | 32 | 1.90 |
| [882470--882821](http://tandem.bu.edu/trf/output/45aYwQQncxAmY.2.7.7.80.10.50.500.1.txt.html#882470--882821,78,4.5,77,80) | 78 | 4.5 | 77 | 76 | 9 | 300 | 34 | 11 | 24 | 30 | 1.90 |
| [882522--882820](http://tandem.bu.edu/trf/output/45aYwQQncxAmY.2.7.7.80.10.50.500.1.txt.html#882522--882820,78,3.8,78,81) | 78 | 3.8 | 78 | 81 | 3 | 305 | 34 | 10 | 24 | 31 | 1.88 |
| [956220--956430](http://tandem.bu.edu/trf/output/45aYwQQncxAmY.2.7.7.80.10.50.500.1.txt.html#956220--956430,111,1.9,111,82) | 111 | 1.9 | 111 | 88 | 0 | 314 | 44 | 10 | 21 | 23 | 1.83 |
| [956590--957401](http://tandem.bu.edu/trf/output/45aYwQQncxAmY.2.7.7.80.10.50.500.1.txt.html#956590--957401,441,1.8,440,83) | 441 | 1.8 | 440 | 80 | 6 | 962 | 41 | 12 | 20 | 25 | 1.87 |
| [967120--967522](http://tandem.bu.edu/trf/output/45aYwQQncxAmY.2.7.7.80.10.50.500.1.txt.html#967120--967522,201,2.0,201,85) | 201 | 2.0 | 201 | 80 | 4 | 456 | 43 | 13 | 20 | 21 | 1.87 |
| [1018001--1018101](http://tandem.bu.edu/trf/output/45aYwQQncxAmY.2.7.7.80.10.50.500.1.txt.html#1018001--1018101,15,6.7,15,86) | 15 | 6.7 | 15 | 77 | 3 | 96 | 64 | 8 | 20 | 5 | 1.43 |
| [1018001--1018111](http://tandem.bu.edu/trf/output/45aYwQQncxAmY.2.7.7.80.10.50.500.1.txt.html#1018001--1018111,45,2.5,45,89) | 45 | 2.5 | 45 | 88 | 4 | 154 | 62 | 9 | 20 | 8 | 1.50 |
| [1018920--1018954](http://tandem.bu.edu/trf/output/45aYwQQncxAmY.2.7.7.80.10.50.500.1.txt.html#1018920--1018954,18,1.9,18,90) | 18 | 1.9 | 18 | 94 | 0 | 61 | 60 | 11 | 25 | 2 | 1.45 |
| [1083579--1083618](http://tandem.bu.edu/trf/output/45aYwQQncxAmY.2.7.7.80.10.50.500.1.txt.html#1083579--1083618,21,1.9,21,91) | 21 | 1.9 | 21 | 89 | 0 | 62 | 30 | 27 | 32 | 10 | 1.89 |
| [1148979--1149019](http://tandem.bu.edu/trf/output/45aYwQQncxAmY.2.7.7.80.10.50.500.1.txt.html#1148979--1149019,22,2.0,22,93) | 22 | 2.0 | 22 | 90 | 9 | 68 | 51 | 12 | 17 | 19 | 1.76 |
| [1181285--1182096](http://tandem.bu.edu/trf/output/45aYwQQncxAmY.2.7.7.80.10.50.500.1.txt.html#1181285--1182096,147,5.5,147,97) | 147 | 5.5 | 147 | 93 | 0 | 1311 | 40 | 14 | 20 | 24 | 1.89 |
| [1216909--1216947](http://tandem.bu.edu/trf/output/45aYwQQncxAmY.2.7.7.80.10.50.500.1.txt.html#1216909--1216947,20,1.9,21,99) | 20 | 1.9 | 21 | 85 | 15 | 55 | 51 | 2 | 12 | 33 | 1.54 |
| [1268347--1269688](http://tandem.bu.edu/trf/output/45aYwQQncxAmY.2.7.7.80.10.50.500.1.txt.html#1268347--1269688,201,6.7,199,100) | 201 | 6.7 | 199 | 83 | 4 | 1347 | 40 | 20 | 15 | 23 | 1.90 |
| [1268347--1269696](http://tandem.bu.edu/trf/output/45aYwQQncxAmY.2.7.7.80.10.50.500.1.txt.html#1268347--1269696,402,3.4,400,101) | 402 | 3.4 | 400 | 82 | 4 | 1508 | 41 | 20 | 15 | 23 | 1.90 |
| [1269342--1269759](http://tandem.bu.edu/trf/output/45aYwQQncxAmY.2.7.7.80.10.50.500.1.txt.html#1269342--1269759,192,2.2,192,102) | 192 | 2.2 | 192 | 89 | 2 | 628 | 42 | 20 | 15 | 22 | 1.89 |
| [1330044--1330107](http://tandem.bu.edu/trf/output/45aYwQQncxAmY.2.7.7.80.10.50.500.1.txt.html#1330044--1330107,33,1.9,33,103) | 33 | 1.9 | 33 | 96 | 0 | 119 | 35 | 9 | 40 | 14 | 1.78 |
| [1330044--1330119](http://tandem.bu.edu/trf/output/45aYwQQncxAmY.2.7.7.80.10.50.500.1.txt.html#1330044--1330119,33,2.3,33,104) | 33 | 2.3 | 33 | 86 | 4 | 91 | 38 | 7 | 39 | 14 | 1.75 |
| [1339358--1339398](http://tandem.bu.edu/trf/output/45aYwQQncxAmY.2.7.7.80.10.50.500.1.txt.html#1339358--1339398,22,1.9,22,105) | 22 | 1.9 | 22 | 85 | 5 | 57 | 51 | 4 | 14 | 29 | 1.63 |
| [1354100--1354180](http://tandem.bu.edu/trf/output/45aYwQQncxAmY.2.7.7.80.10.50.500.1.txt.html#1354100--1354180,27,3.0,27,106) | 27 | 3.0 | 27 | 98 | 0 | 153 | 43 | 37 | 12 | 7 | 1.70 |
| [1372347--1372381](http://tandem.bu.edu/trf/output/45aYwQQncxAmY.2.7.7.80.10.50.500.1.txt.html#1372347--1372381,13,2.6,14,107) | 13 | 2.6 | 14 | 86 | 9 | 54 | 62 | 0 | 2 | 34 | 1.10 |
| [1376631--1376660](http://tandem.bu.edu/trf/output/45aYwQQncxAmY.2.7.7.80.10.50.500.1.txt.html#1376631--1376660,9,3.3,9,108) | 9 | 3.3 | 9 | 95 | 0 | 51 | 40 | 23 | 16 | 20 | 1.91 |
| [1378599--1378668](http://tandem.bu.edu/trf/output/45aYwQQncxAmY.2.7.7.80.10.50.500.1.txt.html#1378599--1378668,33,2.1,33,109) | 33 | 2.1 | 33 | 91 | 0 | 113 | 18 | 11 | 22 | 47 | 1.81 |
| [1380156--1380350](http://tandem.bu.edu/trf/output/45aYwQQncxAmY.2.7.7.80.10.50.500.1.txt.html#1380156--1380350,105,1.9,105,110) | 105 | 1.9 | 105 | 93 | 0 | 336 | 22 | 20 | 28 | 29 | 1.98 |
| [1430996--1431030](http://tandem.bu.edu/trf/output/45aYwQQncxAmY.2.7.7.80.10.50.500.1.txt.html#1430996--1431030,18,2.1,17,113) | 18 | 2.1 | 17 | 89 | 10 | 54 | 28 | 17 | 11 | 42 | 1.83 |
| [1457196--1457561](http://tandem.bu.edu/trf/output/45aYwQQncxAmY.2.7.7.80.10.50.500.1.txt.html#1457196--1457561,42,8.7,42,114) | 42 | 8.7 | 42 | 94 | 0 | 570 | 11 | 20 | 29 | 38 | 1.87 |
| [1507304--1507332](http://tandem.bu.edu/trf/output/45aYwQQncxAmY.2.7.7.80.10.50.500.1.txt.html#1507304--1507332,10,2.9,10,117) | 10 | 2.9 | 10 | 100 | 0 | 58 | 10 | 0 | 17 | 72 | 1.11 |
| [1524999--1525036](http://tandem.bu.edu/trf/output/45aYwQQncxAmY.2.7.7.80.10.50.500.1.txt.html#1524999--1525036,20,2.0,20,118) | 20 | 2.0 | 20 | 89 | 5 | 60 | 60 | 10 | 0 | 28 | 1.30 |
| [1542117--1542157](http://tandem.bu.edu/trf/output/45aYwQQncxAmY.2.7.7.80.10.50.500.1.txt.html#1542117--1542157,12,3.4,12,120) | 12 | 3.4 | 12 | 93 | 0 | 64 | 14 | 14 | 4 | 65 | 1.42 |
| [1542652--1542690](http://tandem.bu.edu/trf/output/45aYwQQncxAmY.2.7.7.80.10.50.500.1.txt.html#1542652--1542690,20,2.1,18,121) | 20 | 2.1 | 18 | 85 | 9 | 51 | 41 | 5 | 7 | 46 | 1.55 |
| [1605267--1605300](http://tandem.bu.edu/trf/output/45aYwQQncxAmY.2.7.7.80.10.50.500.1.txt.html#1605267--1605300,9,3.8,9,122) | 9 | 3.8 | 9 | 96 | 0 | 59 | 0 | 23 | 11 | 64 | 1.26 |
| [1605297--1605344](http://tandem.bu.edu/trf/output/45aYwQQncxAmY.2.7.7.80.10.50.500.1.txt.html#1605297--1605344,9,5.3,9,123) | 9 | 5.3 | 9 | 82 | 0 | 51 | 8 | 14 | 2 | 75 | 1.13 |
| [1605270--1605344](http://tandem.bu.edu/trf/output/45aYwQQncxAmY.2.7.7.80.10.50.500.1.txt.html#1605270--1605344,18,4.2,18,124) | 18 | 4.2 | 18 | 77 | 0 | 60 | 5 | 17 | 6 | 70 | 1.28 |
| [1605288--1605344](http://tandem.bu.edu/trf/output/45aYwQQncxAmY.2.7.7.80.10.50.500.1.txt.html#1605288--1605344,27,2.1,27,125) | 27 | 2.1 | 27 | 80 | 0 | 69 | 7 | 15 | 5 | 71 | 1.25 |
| [1627785--1627818](http://tandem.bu.edu/trf/output/45aYwQQncxAmY.2.7.7.80.10.50.500.1.txt.html#1627785--1627818,15,2.3,15,127) | 15 | 2.3 | 15 | 89 | 0 | 50 | 26 | 14 | 11 | 47 | 1.79 |
| [1641451--1641971](http://tandem.bu.edu/trf/output/45aYwQQncxAmY.2.7.7.80.10.50.500.1.txt.html#1641451--1641971,285,1.8,285,128) | 285 | 1.8 | 285 | 85 | 0 | 729 | 26 | 22 | 19 | 31 | 1.98 |
| [1641451--1642039](http://tandem.bu.edu/trf/output/45aYwQQncxAmY.2.7.7.80.10.50.500.1.txt.html#1641451--1642039,285,2.1,284,133) | 285 | 2.1 | 284 | 82 | 2 | 689 | 25 | 21 | 20 | 32 | 1.97 |
| [1683518--1683806](http://tandem.bu.edu/trf/output/45aYwQQncxAmY.2.7.7.80.10.50.500.1.txt.html#1683518--1683806,159,1.8,156,135) | 159 | 1.8 | 156 | 89 | 3 | 454 | 19 | 12 | 23 | 44 | 1.85 |
| [1728136--1728210](http://tandem.bu.edu/trf/output/45aYwQQncxAmY.2.7.7.80.10.50.500.1.txt.html#1728136--1728210,33,2.3,33,136) | 33 | 2.3 | 33 | 81 | 4 | 89 | 8 | 20 | 25 | 46 | 1.77 |
| [1728355--1728400](http://tandem.bu.edu/trf/output/45aYwQQncxAmY.2.7.7.80.10.50.500.1.txt.html#1728355--1728400,22,2.0,24,137) | 22 | 2.0 | 24 | 83 | 8 | 60 | 0 | 15 | 39 | 45 | 1.46 |
| [1730701--1730726](http://tandem.bu.edu/trf/output/45aYwQQncxAmY.2.7.7.80.10.50.500.1.txt.html#1730701--1730726,12,2.2,12,138) | 12 | 2.2 | 12 | 100 | 0 | 52 | 23 | 15 | 15 | 46 | 1.83 |
| [1742398--1742438](http://tandem.bu.edu/trf/output/45aYwQQncxAmY.2.7.7.80.10.50.500.1.txt.html#1742398--1742438,21,2.0,21,139) | 21 | 2.0 | 21 | 85 | 9 | 57 | 19 | 19 | 12 | 48 | 1.80 |
| [1790612--1790639](http://tandem.bu.edu/trf/output/45aYwQQncxAmY.2.7.7.80.10.50.500.1.txt.html#1790612--1790639,12,2.3,12,140) | 12 | 2.3 | 12 | 100 | 0 | 56 | 50 | 28 | 14 | 7 | 1.69 |
| [1801693--1801868](http://tandem.bu.edu/trf/output/45aYwQQncxAmY.2.7.7.80.10.50.500.1.txt.html#1801693--1801868,42,4.2,42,141) | 42 | 4.2 | 42 | 87 | 1 | 219 | 55 | 12 | 24 | 7 | 1.64 |
| [1863188--1863606](http://tandem.bu.edu/trf/output/45aYwQQncxAmY.2.7.7.80.10.50.500.1.txt.html#1863188--1863606,225,1.9,225,143) | 225 | 1.9 | 225 | 89 | 1 | 660 | 22 | 19 | 17 | 40 | 1.91 |
| [1863188--1863690](http://tandem.bu.edu/trf/output/45aYwQQncxAmY.2.7.7.80.10.50.500.1.txt.html#1863188--1863690,225,2.2,224,145) | 225 | 2.2 | 224 | 85 | 2 | 616 | 22 | 20 | 16 | 40 | 1.91 |
| [1871310--1871480](http://tandem.bu.edu/trf/output/45aYwQQncxAmY.2.7.7.80.10.50.500.1.txt.html#1871310--1871480,78,2.2,78,147) | 78 | 2.2 | 78 | 87 | 0 | 243 | 29 | 20 | 13 | 36 | 1.91 |
| [1871979--1872102](http://tandem.bu.edu/trf/output/45aYwQQncxAmY.2.7.7.80.10.50.500.1.txt.html#1871979--1872102,39,3.2,39,148) | 39 | 3.2 | 39 | 62 | 21 | 96 | 29 | 23 | 13 | 33 | 1.93 |
| [1871921--1872341](http://tandem.bu.edu/trf/output/45aYwQQncxAmY.2.7.7.80.10.50.500.1.txt.html#1871921--1872341,78,5.4,78,149) | 78 | 5.4 | 78 | 88 | 2 | 479 | 29 | 22 | 13 | 34 | 1.92 |
| [1872174--1872302](http://tandem.bu.edu/trf/output/45aYwQQncxAmY.2.7.7.80.10.50.500.1.txt.html#1872174--1872302,39,3.3,39,151) | 39 | 3.3 | 39 | 64 | 16 | 104 | 31 | 24 | 10 | 34 | 1.90 |
| [1872213--1872336](http://tandem.bu.edu/trf/output/45aYwQQncxAmY.2.7.7.80.10.50.500.1.txt.html#1872213--1872336,39,3.2,39,152) | 39 | 3.2 | 39 | 66 | 21 | 105 | 31 | 24 | 11 | 33 | 1.90 |
| [1872783--1873052](http://tandem.bu.edu/trf/output/45aYwQQncxAmY.2.7.7.80.10.50.500.1.txt.html#1872783--1873052,78,3.5,77,156) | 78 | 3.5 | 77 | 85 | 3 | 326 | 32 | 22 | 10 | 34 | 1.88 |
| [1873506--1873805](http://tandem.bu.edu/trf/output/45aYwQQncxAmY.2.7.7.80.10.50.500.1.txt.html#1873506--1873805,156,1.9,156,159) | 156 | 1.9 | 156 | 86 | 1 | 431 | 31 | 21 | 12 | 35 | 1.90 |
| [1873661--1873784](http://tandem.bu.edu/trf/output/45aYwQQncxAmY.2.7.7.80.10.50.500.1.txt.html#1873661--1873784,39,3.2,39,160) | 39 | 3.2 | 39 | 69 | 13 | 128 | 35 | 20 | 8 | 36 | 1.82 |
| [1873506--1873835](http://tandem.bu.edu/trf/output/45aYwQQncxAmY.2.7.7.80.10.50.500.1.txt.html#1873506--1873835,78,4.2,77,164) | 78 | 4.2 | 77 | 83 | 3 | 358 | 31 | 20 | 12 | 35 | 1.90 |
| [1912606--1912828](http://tandem.bu.edu/trf/output/45aYwQQncxAmY.2.7.7.80.10.50.500.1.txt.html#1912606--1912828,121,1.8,122,166) | 121 | 1.8 | 122 | 80 | 8 | 267 | 31 | 14 | 21 | 31 | 1.94 |
| [1941104--1941199](http://tandem.bu.edu/trf/output/45aYwQQncxAmY.2.7.7.80.10.50.500.1.txt.html#1941104--1941199,39,2.5,39,167) | 39 | 2.5 | 39 | 84 | 3 | 122 | 23 | 27 | 11 | 37 | 1.89 |
| [1941104--1941214](http://tandem.bu.edu/trf/output/45aYwQQncxAmY.2.7.7.80.10.50.500.1.txt.html#1941104--1941214,39,2.8,39,168) | 39 | 2.8 | 39 | 82 | 2 | 107 | 22 | 27 | 13 | 36 | 1.92 |
| [1962954--1963000](http://tandem.bu.edu/trf/output/45aYwQQncxAmY.2.7.7.80.10.50.500.1.txt.html#1962954--1963000,24,2.0,24,169) | 24 | 2.0 | 24 | 83 | 8 | 60 | 29 | 17 | 6 | 46 | 1.72 |
| [1991055--1991081](http://tandem.bu.edu/trf/output/45aYwQQncxAmY.2.7.7.80.10.50.500.1.txt.html#1991055--1991081,10,2.7,10,171) | 10 | 2.7 | 10 | 100 | 0 | 54 | 0 | 7 | 18 | 74 | 1.05 |
| [2011134--2011980](http://tandem.bu.edu/trf/output/45aYwQQncxAmY.2.7.7.80.10.50.500.1.txt.html#2011134--2011980,228,3.7,228,172) | 228 | 3.7 | 228 | 96 | 0 | 1511 | 21 | 18 | 19 | 40 | 1.92 |
| [2013432--2013461](http://tandem.bu.edu/trf/output/45aYwQQncxAmY.2.7.7.80.10.50.500.1.txt.html#2013432--2013461,15,1.9,16,173) | 15 | 1.9 | 16 | 93 | 6 | 53 | 33 | 13 | 10 | 43 | 1.77 |
| [2014268--2014292](http://tandem.bu.edu/trf/output/45aYwQQncxAmY.2.7.7.80.10.50.500.1.txt.html#2014268--2014292,9,2.8,9,174) | 9 | 2.8 | 9 | 100 | 0 | 50 | 0 | 12 | 20 | 68 | 1.21 |
| [2014353--2014646](http://tandem.bu.edu/trf/output/45aYwQQncxAmY.2.7.7.80.10.50.500.1.txt.html#2014353--2014646,153,1.9,153,175) | 153 | 1.9 | 153 | 81 | 4 | 351 | 24 | 18 | 19 | 38 | 1.93 |
| [2051485--2051514](http://tandem.bu.edu/trf/output/45aYwQQncxAmY.2.7.7.80.10.50.500.1.txt.html#2051485--2051514,15,2.0,15,179) | 15 | 2.0 | 15 | 100 | 0 | 60 | 20 | 13 | 6 | 60 | 1.55 |
| [2087939--2087964](http://tandem.bu.edu/trf/output/45aYwQQncxAmY.2.7.7.80.10.50.500.1.txt.html#2087939--2087964,13,2.0,13,180) | 13 | 2.0 | 13 | 100 | 0 | 52 | 15 | 30 | 0 | 53 | 1.42 |
| [2093771--2094058](http://tandem.bu.edu/trf/output/45aYwQQncxAmY.2.7.7.80.10.50.500.1.txt.html#2093771--2094058,78,3.7,78,182) | 78 | 3.7 | 78 | 81 | 3 | 280 | 33 | 24 | 10 | 31 | 1.89 |
| [2093758--2094087](http://tandem.bu.edu/trf/output/45aYwQQncxAmY.2.7.7.80.10.50.500.1.txt.html#2093758--2094087,78,4.2,78,184) | 78 | 4.2 | 78 | 77 | 8 | 266 | 31 | 23 | 11 | 33 | 1.90 |
| [2095162--2095664](http://tandem.bu.edu/trf/output/45aYwQQncxAmY.2.7.7.80.10.50.500.1.txt.html#2095162--2095664,78,6.5,76,185) | 78 | 6.5 | 76 | 77 | 10 | 333 | 33 | 22 | 10 | 34 | 1.88 |
| [2095245--2095367](http://tandem.bu.edu/trf/output/45aYwQQncxAmY.2.7.7.80.10.50.500.1.txt.html#2095245--2095367,39,3.2,39,186) | 39 | 3.2 | 39 | 64 | 17 | 101 | 34 | 19 | 10 | 34 | 1.86 |
| [2095228--2095731](http://tandem.bu.edu/trf/output/45aYwQQncxAmY.2.7.7.80.10.50.500.1.txt.html#2095228--2095731,156,3.2,154,187) | 156 | 3.2 | 154 | 76 | 7 | 450 | 33 | 22 | 9 | 34 | 1.87 |
| [2162010--2162468](http://tandem.bu.edu/trf/output/45aYwQQncxAmY.2.7.7.80.10.50.500.1.txt.html#2162010--2162468,119,3.9,119,188) | 119 | 3.9 | 119 | 75 | 9 | 443 | 33 | 15 | 20 | 30 | 1.93 |
| [2246790--2246822](http://tandem.bu.edu/trf/output/45aYwQQncxAmY.2.7.7.80.10.50.500.1.txt.html#2246790--2246822,11,3.0,11,189) | 11 | 3.0 | 11 | 95 | 0 | 57 | 48 | 9 | 15 | 27 | 1.74 |
| [2390929--2390960](http://tandem.bu.edu/trf/output/45aYwQQncxAmY.2.7.7.80.10.50.500.1.txt.html#2390929--2390960,16,2.0,16,190) | 16 | 2.0 | 16 | 93 | 0 | 55 | 6 | 21 | 6 | 65 | 1.38 |
| [2477936--2478006](http://tandem.bu.edu/trf/output/45aYwQQncxAmY.2.7.7.80.10.50.500.1.txt.html#2477936--2478006,33,2.2,33,191) | 33 | 2.2 | 33 | 89 | 5 | 108 | 49 | 22 | 5 | 22 | 1.71 |
| [2477951--2478006](http://tandem.bu.edu/trf/output/45aYwQQncxAmY.2.7.7.80.10.50.500.1.txt.html#2477951--2478006,15,3.5,15,192) | 15 | 3.5 | 15 | 75 | 17 | 51 | 48 | 23 | 7 | 21 | 1.74 |
| [2477936--2477991](http://tandem.bu.edu/trf/output/45aYwQQncxAmY.2.7.7.80.10.50.500.1.txt.html#2477936--2477991,18,3.3,18,194) | 18 | 3.3 | 18 | 69 | 23 | 57 | 50 | 21 | 5 | 23 | 1.69 |
| [2477961--2478010](http://tandem.bu.edu/trf/output/45aYwQQncxAmY.2.7.7.80.10.50.500.1.txt.html#2477961--2478010,15,3.1,15,195) | 15 | 3.1 | 15 | 76 | 15 | 55 | 50 | 24 | 6 | 20 | 1.70 |
| [2523242--2523485](http://tandem.bu.edu/trf/output/45aYwQQncxAmY.2.7.7.80.10.50.500.1.txt.html#2523242--2523485,122,2.0,121,196) | 122 | 2.0 | 121 | 89 | 0 | 371 | 30 | 22 | 13 | 33 | 1.93 |
| [2701934--2701971](http://tandem.bu.edu/trf/output/45aYwQQncxAmY.2.7.7.80.10.50.500.2.txt.html#2701934--2701971,12,3.2,12,197) | 12 | 3.2 | 12 | 88 | 0 | 58 | 23 | 7 | 15 | 52 | 1.69 |
| [2702122--2702187](http://tandem.bu.edu/trf/output/45aYwQQncxAmY.2.7.7.80.10.50.500.2.txt.html#2702122--2702187,33,2.0,33,198) | 33 | 2.0 | 33 | 84 | 0 | 87 | 21 | 12 | 24 | 42 | 1.86 |
| [2834651--2834696](http://tandem.bu.edu/trf/output/45aYwQQncxAmY.2.7.7.80.10.50.500.2.txt.html#2834651--2834696,15,3.1,15,199) | 15 | 3.1 | 15 | 90 | 0 | 74 | 0 | 21 | 8 | 69 | 1.15 |
| [2834649--2834710](http://tandem.bu.edu/trf/output/45aYwQQncxAmY.2.7.7.80.10.50.500.2.txt.html#2834649--2834710,21,3.0,20,200) | 21 | 3.0 | 20 | 75 | 17 | 63 | 3 | 20 | 8 | 67 | 1.31 |
| [2844707--2844734](http://tandem.bu.edu/trf/output/45aYwQQncxAmY.2.7.7.80.10.50.500.2.txt.html#2844707--2844734,6,4.7,6,201) | 6 | 4.7 | 6 | 100 | 0 | 56 | 32 | 17 | 50 | 0 | 1.47 |
| [2845149--2845714](http://tandem.bu.edu/trf/output/45aYwQQncxAmY.2.7.7.80.10.50.500.2.txt.html#2845149--2845714,273,2.1,273,203) | 273 | 2.1 | 273 | 98 | 0 | 1087 | 20 | 19 | 20 | 39 | 1.93 |
| [2846191--2846219](http://tandem.bu.edu/trf/output/45aYwQQncxAmY.2.7.7.80.10.50.500.2.txt.html#2846191--2846219,15,1.9,15,204) | 15 | 1.9 | 15 | 100 | 0 | 58 | 10 | 6 | 20 | 62 | 1.50 |
| **Plasmid** |  |  |  |  |  |  |  |  |  |  |  |
| [13917--13942](http://tandem.bu.edu/trf/output/46oALyU8DD4vg.2.7.7.80.10.50.500.1.txt.html#13917--13942,8,3.3,8,1) | 8 | 3.3 | 8 | 100 | 0 | 52 | 76 | 0 | 23 | 0 | 0.78 |
| [13972--13999](http://tandem.bu.edu/trf/output/46oALyU8DD4vg.2.7.7.80.10.50.500.1.txt.html#13972--13999,8,3.5,8,2) | 8 | 3.5 | 8 | 100 | 0 | 56 | 78 | 0 | 21 | 0 | 0.75 |
